# Temporal Ordering of Inflammatory Analytes sTNFR2 and sTREM2 in Relation to Alzheimer's Disease Biomarkers and Clinical Outcomes

**DOI:** 10.3389/fnagi.2021.676744

**Published:** 2021-06-29

**Authors:** Jagan A. Pillai, Maria Khrestian, James Bena, James B. Leverenz, Lynn M. Bekris

**Affiliations:** ^1^Department of Neurology, Cleveland Clinic, Cleveland, OH, United States; ^2^Lou Ruvo Center for Brain Health, Cleveland Clinic, Cleveland, OH, United States; ^3^Neurological Institute, Cleveland Clinic, Cleveland, OH, United States; ^4^Genomic Medicine Institute, Cleveland Clinic, Cleveland, OH, United States; ^5^Lerner Research Institute, Cleveland Clinic, Cleveland, OH, United States; ^6^Quantitative Health Sciences, Cleveland Clinic, Cleveland, OH, United States

**Keywords:** Alzheimer's disease, soluble TREM2, soluble TNFR2, inflammation, MCI, dementia, preclinical AD, ATN classification

## Abstract

Inflammatory changes are among the key markers of Alzheimer's disease (AD) related pathological changes. Pro-inflammatory analytes have been related to cognitive decline while others have been related to attenuating neuronal death. Among them, changes in cerebrospinal fluid (CSF) levels of soluble triggering receptor expressed on myeloid cells 2 (sTREM2) and soluble tumor necrosis factor receptor 2 (sTNFR2) have been described as impacting favorable clinical outcomes in AD. We therefore evaluate the effect of CSF sTREM2 and sTNFR2 when taken together on AD biomarkers and longitudinal clinical decline to understand their relative role on impacting AD clinical biomarkers and subsequent clinical outcomes. This longitudinal observational cohort study included 168 amyloid-positive (A+) and p-tau-positive (T+) participants with mild cognitive impairment (MCI) or AD dementia from the Alzheimer's Disease Neuroimaging Initiative (ADNI) with 109 of them having concomitant CSF sTREM2 and sTNFR2 data and 48 A+ T+ participants with MCI from a tertiary memory clinic cohort. An exploratory analysis was performed using data from 86 cognitively normal (CN) participants from ADNI with 72 of them having concomitant CSF AD biomarkers and CSF sTREM2 and sTNFR2 data. General linear models were used to evaluate the effect of sTREM2 and sTNFR2 levels on baseline CSF Aβ42, t-tau, and p-tau, and a linear mixed-effects model was used to assess longitudinal cognitive change after controlling for well-known covariates. Among ADNI A+ T+ MCI and AD dementia participants, CSF sTNFR2 had a stronger association, than CSF sTREM2, with CSF t-tau and p-tau. This was replicated among A+ T+ MCI participants from the memory clinic cohort. On the contrary, among A+ T+ CN participants, CSF sTREM2 explained significant variance in CSF t-tau and p-tau, while CSF sTNFR2 did not. When the effects of CSF sTNFR2 and t-tau on longitudinal cognitive change were taken into account, higher CSF sTREM2 predicted slower cognitive decline in A+ T+ AD dementia participants and faster decline in A+ T+ CN participants. Our results show that given the dynamic changes in sTREM2 and sTNFR2, the clinical impact of these distinct inflammation related biomarkers in tracking AD temporal progression across disease stages are likely to differ.

## Background

Genome-wide association studies in Alzheimer's disease (AD) have noted multiple susceptibility loci for late-onset AD related to the innate immune system (Lambert et al., 2013; Van Cauwenberghe et al., 2016). Among these susceptibility loci, the presence of the gene encoding the triggering receptor expressed on myeloid cells 2 (*TREM2*) has been reported to increase the risk of AD development by 2–3-fold (Guerreiro et al., 2013; Jonsson et al., 2013). *TREM2* promotes anti-inflammatory cytokine expression, reduces pro-inflammatory cytokine release, and is involved in osteoclast development and the activation of brain microglia and monocyte-derived dendritic cells (Carmona et al., 2018). *TREM2* is also thought to enhance the rate of phagocytosis and to modulate inflammatory signaling (Gratuze et al., 2018).

These results, amongst animal and *in vitro* models, have prompted researchers to evaluate how *TREM2* may mediate clinical outcomes of interest in AD. However, the results of these studies have been more nuanced with regard to *TREM's* effects in clinical AD. For instance, network analysis of post-mortem AD brain gene expression with the highest connectivity to *TREM2* revealed both anti- and pro-inflammatory gene clusters (Forabosco et al., 2013). Clinical reports regarding cerebrospinal fluid (CSF) levels of sTREM2, a soluble TREM2 protein fragment produced by the cleavage of TREM2, have demonstrated varying levels of this protein in the different stages of AD (Wunderlich et al., 2013). Although most studies have shown that CSF sTREM2 is increased in the presence of AD biomarkers, the results are somewhat inconsistent regarding sTREM2 levels in amyloid-positive (A+) and tau-positive (T+) cognitively normal (CN) individuals (Ewers et al., 2019; Suárez-Calvet et al., 2019), patients with mild cognitive impairment (MCI) (Gispert et al., 2016b; Henjum et al., 2016; Suárez-Calvet et al., 2016b, 2019; Ewers et al., 2019; Knapskog et al., 2020), and those with AD dementia (Gispert et al., 2016b; Piccio et al., 2016; Suárez-Calvet et al., 2016b, 2019). Some studies have demonstrated no differences in sTREM2 levels across the AD spectrum (Gispert et al., 2016a; Henjum et al., 2016; Knapskog et al., 2020), whereas other research has demonstrated decreased levels of sTREM2 in patients with AD dementia, perhaps partially reflecting the variability in clinical symptoms even within the same stage of AD (Kleinberger et al., 2014; Bekris et al., 2018). However, other studies have found that CSF sTREM2 has a dynamic response in the tracking of AD progression (Suárez-Calvet et al., 2016c, 2019; Ma et al., 2020), and a study in patients with MCI or AD dementia who had A+ and T+ biomarkers found that higher concentrations of sTREM2 in CSF were associated with reduced memory decline, lower CSF p-tau levels, and hippocampal shrinkage (Ewers et al., 2019).

A variety of other inflammatory analytes in the CSF are altered in both pre-symptomatic (Janelidze et al., 2018) and subsequent clinical stages of AD (Pillai et al., 2019b). While pro-inflammatory analyte levels in the CSF have been noted to predict AD disease progression (Pillai et al., 2020), other inflammatory markers have been reported to attenuate neuronal death and affect clinical outcomes favorably in AD including sTREM2 (Ewers et al., 2019; Chen et al., 2020; Franzmeier et al., 2020). Recently, our group found that the inflammatory gene, *TNFRSF1B* and related soluble tumor necrosis factor receptor 2 (sTNFR2) CSF levels also relate to favorable clinical outcomes in AD (Pillai et al., 2021). TNFR2 is thought to promote downstream antiapoptotic responses and to play a protective role against neurodegeneration (Fischer et al., 2011; Dong et al., 2016). The effect of TREM2 levels on AD biomarkers and clinical outcomes has garnered significant interest as detailed earlier but there are limited studies of the effects of TNFR2 on AD biomarkers and on clinical outcomes in different stages of AD.

The potential interaction between *TNFRSF1B* and *TREM2* has also been the focus of recent research in animal models. In a study of *TNFRSF1B* conditional knockout mice, lack of TNFR2 activation was found to impair constitutive expression and transcriptional regulation of *TREM2* by soluble TNF (Gao et al., 2017). These findings suggest a complex interplay across the inflammation-related pathways underlying neurodegeneration, making clinical studies of this subject challenging but critical. Elucidating the relationship between sTREM2 and sTNFR2 and the effect of these analytes on AD biomarkers and patient outcomes could help us to make more effective use of these analytes as clinical biomarkers, as well as develop therapeutic strategies that target these inflammation-related pathways.

We therefore sought to evaluate CSF sTREM2 and CSF sTNFR2 levels across the continuum of AD, and to determine the relationship between these analyte levels and AD CSF biomarkers and longitudinal cognitive outcomes. We used data from the Alzheimer's Disease Neuroimaging Initiative (ADNI) research cohort to test whether CSF sTREM2 levels correlate with sTNFR2 among MCI and dementia subjects classified as AD, aggregated Aβ (A+) and aggregated tau (T+), according to the National Institute on Aging and Alzheimer's Association (NIA-AA) AT(N) framework (Jack et al., 2018) and whether CSF sTREM2 levels independent of sTNFR2 levels are associated with the AD biomarkers CSF Aβ42, t-tau, and p-tau. We evaluated the reliability of these results in ADNI by assessing the same variables among A+ T+ MCI participants being treated at a memory clinic. We also sought to evaluate whether CSF sTREM2 levels, independent of sTNFR2, are associated with favorable clinical outcomes in AD. Based on previous reports (Ewers et al., 2019; Franzmeier et al., 2020), we hypothesized that higher concentrations of sTREM2 in CSF would be associated with slower rates of cognitive decline in both the A+ T+ MCI and A+ T+ dementia stages of AD. Finally, we explored the consistency of these results in a smaller cohort of cognitively normal (CN) individuals from the ADNI cohort who met A– T–, A– T+, A+ T–, or A+ T+ criteria.

## Materials and Methods

### Study Cohort: ADNI

The ADNI is a longitudinal multicenter study designed to develop clinical, imaging, genetic, and biochemical biomarkers for the early detection and tracking of AD. ADNI was launched by the National Institute of Aging with additional support from private pharmaceutical companies and non-profit organizations. The eligibility criteria for the first phase of the ADNI study are described in the ADNI1 protocol (http://adni.loni.usc.edu/methods/documents/). Briefly, eligible participants were aged 55–90 years, had an informant able to provide an independent evaluation of functioning, and spoke either English or Spanish. Participants had completed at least 6 years of education (or had a work history sufficient to exclude intellectual disability). For clinical staging, the categories of CN, MCI, and AD dementia were used (Jack et al., 2011, 2018).

Details regarding the Elecsys method used to measure AD biomarkers in the ADNI cohort are described elsewhere (Shaw et al., 2019). Following the ATN criteria, amyloid deposition (A+) was defined as abnormal values of CSF Aβ1–42, and tau pathology (T+) was defined as abnormal values of CSF p-tau181 (Jack et al., 2017, 2018). Based on previously published cut points in the ADNI sample (Ewers et al., 2019), the criterion for A+ was defined as Aβ1–42 < 976.6 pg/mL; the criterion for T+ was defined as p-tau181 > 21.8 pg/mL. A total of 109 participants in ADNI (MCI, *n* = 67; AD dementia, *n* = 42) met the A+ T+ criteria at baseline and had data on CSF sTREM2 and CSF sTNFR2 (Table 1).

**Table 1:** Demographics of participants from the Alzheimer's Disease Neuroimaging Initiative (ADNI) and replication memory clinic cohorts (participants meeting A+ T+ criteria and concomitant sTREM2 and sTNFR2 data).

**Demographic variable**	**ADNI MCI cohort (*n* = 67)**	**ADNI AD dementia cohort (*n* = 42)**	**Replication MCI memory clinic cohort (*n* = 48)**	***P*-value^a^**
	**Mean (SD)**	**Mean (SD)**	**Mean (SD)**	
Age, y	74.06 (6.99)^1^	74.16 (7.87)^2^	68.10 (7.3)^1^^,^^2^	<0.0001
Sex (% female)	41.8%	47.6%	41.7%	0.49
*APOEε4* (%)	71.6%	80.9%	77.1%	<0.0001
Patient education, y	15.75 (3.01)	15.17 (3.02)	15.37 (2.87)	0.58
Baseline MMSE score	26.75 (1.76)^3^^,^^4^	23.45 (2.00)^3^^,^^5^	24.8 (3.1)^4^^,^^5^	<0.0001
Baseline CDR-SB score	1.59 (0.9)^6^^,^^7^	4.22 (1.51)^6^^,^^8^	2.17 (1.2)^7^^,^^8^	<0.0001
Log_2_ CSF Aβ42^b^	9.21 (0.39)	9.06 (0.45)	8.12 (0.55)	
Log_2_ CSF t-tau^b^	8.48 (0.38)	8.50 (0.40)	8.93 (0.92)	
Log_2_ CSF p-tau^b^	5.17 (0.43)	5.19 (0.45)	6.27 (0.67)	
Log_2_ CSF sTREM2	12.0 (0.69)^9^	12.0 (0.66)^10^	10.26 (0.75)^c^^,^^9^^,^^10^	<0.0001
Log_2_ CSF sTNFR2	−0.12 (0.15)^11^	−0.11 (0.15)^12^	1.15 (0.45)^11^^,^^12^	<0.0001
Years of follow up	5.0 (2.5)	3.0 (0.58)	—^d^	<0.0001

*AD, Alzheimer's disease; CDR-SB, Clinical Dementia Rating–Sum of Boxes; MCI, mild cognitive impairment; MMSE, Mini-Mental State Exam; SD, standard deviation; sTNFR2, soluble tumor necrosis factor receptor 2; sTREM2, soluble triggering receptor expressed on myeloid cells 2*.

a *P-values from ANOVA for continuous variables and from χ^2^ tests for categorical variables*.

b *CSF Aβ, t-tau, and p-tau levels measured in ADNI by Elecsys method and in replication cohort by INNOTEST ELISA*.

c *n = 42*.

d *only baseline data analyzed. Tukey HSD Post–hoc test:*

1 *ADNI AD MCI vs. Replication AD MCI: Diff = −5.9600, p = 0.0001*.

2 *ADNI AD dementia vs. Replication AD MCI: Diff = −6.0600, p = 0.0004*.

3 *ADNI AD MCI vs. ADNI AD dementia: Diff = −3.3000, p < 0.0001*.

4 *ADNI AD MCI vs. Replication AD MCI: Diff = −1.9500, p ≤ 0.0001*.

5 *ADNI AD dementia vs. Replication AD MCI: Diff = 1.3500, p = 0.017*.

6 *ADNI AD MCI vs. ADNI AD dementia: Diff = 2.6300, p < 0.0001*.

7 *ADNI AD MCI vs. Replication AD MCI: Diff = 0.5800, p = 0.027*.

8 *ADNI AD dementia vs. Replication AD MCI: Diff = 2.0500, p = 0.00001*.

9 *ADNI AD MCI vs. Replication AD MCI: Diff = −1.7400, p < 0.0001*.

10 *ADNI AD dementia vs. Replication AD MCI: Diff = −1.7400, p < 0.0001*.

11 *ADNI AD MCI vs. Replication AD MCI: Diff = 1.2700, p < 0.0001*.

12 *ADNI AD dementia vs. Replication AD MCI: Diff = 1.2600, p < 0.0001*.

#### CSF sTNFR2 Levels and CSF sTREM2 Levels in ADNI

In ADNI, levels of sTNFR2 are measured in CSF samples using the RBM DiscoveryMAP® v.1.0 panel, which uses a Luminex platform (Myriad Genetics; Salt Lake City, UT). The CSF multiplex data used in this analysis were cleaned and quality controlled based on methodology described in the statistical analysis of the Biomarkers Consortium data primer^1^.

The CSF sTREM2 assay used in ADNI is based on the Meso Scale Diagnostics platform and has been described previously (Suárez-Calvet et al., 2016b; Ewers et al., 2019). The CSF sTREM2 values used in this study were corrected based on the values of the four internal standards that were loaded on all plates (variable “MSD_sTREM2CORRECTED” in the ADNI database). Further details regarding the CSF sTREM2 measurements in the ADNI samples, as well as the original data, are available at https://ida.loni.usc.edu.

#### Cognitive and Functional Measures

The Mini-Mental State Exam (MMSE) (Folstein et al., 1975) and Clinical Dementia Rating–Sum of Boxes (CDR-SB) (Morris, 1993) were used to characterize the degree of baseline cognitive and functional deficits. CDR-SB scores were also evaluated longitudinally to assess cognitive change from baseline.

### Study Cohort: Replication Memory Clinic

A cross-sectional replication cohort was created, including 48 participants in the MCI stage of AD (MCI-AD) who were recruited from a specialized memory clinic at Cleveland Clinic (Lou Ruvo Center for Brain Health, Cleveland site). Recruitment details have been described previously (Pillai et al., 2019b, 2020). In brief, consent was obtained from participants to include their CSF, plasma, and DNA samples in the Lou Ruvo Center for Brain Health Aging and Neurodegeneration Biobank (CBH-Biobank), following approval by the local Institutional Review Board.

In these participants, the diagnosis of MCI-AD was confirmed by the presence of CSF Aβ42 and p-tau levels consistent with a diagnosis of AD as the primary etiology; the diagnosis was also confirmed by two neurologists (JP, JL) using published criteria (A+ T+) (Albert et al., 2011). A commercially available test (ADmark® Alzheimer's Evaluation, Athena Diagnostics; Marlborough, MA) was used to measure CSF levels of Aβ42, t-tau, and p-tau. The ADmark® Alzheimer's evaluation uses sandwich Enzyme Linked Immunosorbant Assay (ELISA) kits [Innotest β-amyloid[1–42], Innotest hTAU-Ag, Innotest Phospho-Tau[181P], Innogenetics, Ghent, Belgium]. All participants met the cutoff of Aβ42 ≤ 530 pg/mL, which is consistent with A+ status on the Amyvid TM (Florbetapir F 18 Injection; Eli Lilly and Company; Indianapolis, IN) positron emission tomography used at our center. Participants also met the diagnostic threshold for p-tau per the ADmark test, ≥60 pg/mL consistent with a T+ status. *APOE* status was determined through assessment of blood samples (10 ng per patient) dispensed into 96-well plates for TaqMan (Thermo Fisher Scientific, Waltham, MA) allelic discrimination detection of single nucleotide polymorphisms that discriminate the *APOE* alleles (*rs429358, rs7412*). Polymerase chain reaction (PCR) was performed using a 9700 Gene Amp PCR system (Applied Biosystems, Waltham, MA) and an end-point read in a 7500 Real-Time PCR system (Applied Biosystems).

#### CSF sTNFR2 and sTREM2 Levels in the Replication Memory Clinic

Details regarding CSF sampling for sTNFR2 have been published previously (Pillai et al., 2021). In brief, CSF was collected and analyzed by an independent laboratory via the validated RBM Multi-Analyte Profile (MAP) platform from Myriad Genetics. The RBM HumanMAP® v.2.0 used in the replication cohort is a subset of the RBM DiscoveryMAP® v.1.0 used in ADNI with the same quality control and thresholding process. The least detectable dose of sTNFR2 was 0.0017 ng/L. Samples were frozen within 15 min of collection, were processed at −70°C (in dry ice), and were continuously maintained at −80°C (in a maximum non-frost-free–type refrigerator). The samples were shipped frozen in a Styrofoam container with sufficient dry ice to maintain the temperature below −70°C for at least 48 h. Samples therefore underwent a single freeze-thaw cycle before analysis.

The CSF sTREM2 assay used in the Bekris lab has been described previously (Bekris et al., 2018). In brief, CSF sTREM2 levels were measured using a Luminex 200 3.1 xPONENT System (EMD Millipore; Chicago, IL) and a custom-designed detection method to capture sTREM2. With this method, a capture antibody bound to MagPlex beads binds sTREM2 (R&D #MAB1828 human TREM2 antibody monoclonal mouse IgG2B Clone #263602; Immunogen His19-Ser174), and a biotinylated antibody with a SAPE conjugate is then used for detection (R&D: #BAF1828; human TREM2 biotinylated antibody; antigen affinity-purified polyclonal goat IgG; Immunogen His19-Ser174).

#### Cognitive and Functional Measures

As in the ADNI cohort, the MMSE and CDR-SB were used to characterize the degree of baseline cognitive and functional deficits in the replication memory clinic cohort. CDR-SB scores were also evaluated longitudinally to assess cognitive change from baseline.

### Exploratory Analysis in ADNI CN Participants

CN participants as defined in the ADNIMERGE dataset (downloaded on May 6, 2020) with concomitant data on CSF sTREM2 and CSF sTNFR were included in the exploratory analysis (*n* = 72). Again following the ATN criteria, amyloid deposition (A+) was defined as an abnormal value of CSF Aβ1–42, and tau pathology (T+) was defined as an abnormal value of CSF p-tau181 (Shaw et al., 2019). The cutoff points were as described earlier for the MCI and AD dementia groups (Jack et al., 2017). At baseline, 35 CN participants with sTREM2 and sTNFR2 data were A– T–, 14 were A+ T+, 11 were A+ T–, and 12 were A– T+ (Supplementary Table 1).

Additionally, a sensitivity analysis to evaluate the robustness of results was repeated among participants that had data on CSF sTREM2 (including those lacking concomitant CSF sTNFR2 data) and AD biomarkers (A+T+ MCI, *n* = 111, A = T+ AD dementia, *n* = 57) (Supplementary Table 11) and 86 cognitively normal participants with CSF sTREM2 values (including those lacking concomitant sTNFR2 data) (Supplementary Table 12).

### Statistical Analysis

A log (base 2) transformation allowed Pearson correlations to be fit for exploratory univariate analyses for all analytes, and the levels described are therefore dimensionless. Normality of biomarkers was evaluated using Shapiro-Wilk tests and graphical methods. Pearson estimates of correlation and *P*-values were calculated for sTREM2, sTNFR2, and AD biomarkers. All tests were two-tailed, with the significance level set at 0.05. Sensitivity analyses were completed to assess the robustness of the effects, and collinearity between sTNFR2 and sTREM2 was assessed for the dependent variables t-tau, p-tau, and Aβ42. IBM SPSS Statistics for Windows, version 22.0 (Armonk, NY), and R Core Team RStudio (version 1.2.5042) were used for all analyses.

#### Model 1

To evaluate the effect of sTREM2 and sTNFR2 individually, multivariate general linear models were used to assess the effect of baseline CSF sTREM2 or sTNFR2 on CSF t-tau, p-tau, and Aβ42 levels (dependent variable) after controlling for well-known covariates of age, sex, education years, and *APOE*ε*4* status. Effect size was calculated using partial η^2^. F-test for lack-of-fit and residual plots were used to assess the model linearity and fit.

#### Model 2

To evaluate the effect of sTREM2 and sTNFR2 together, multivariate general linear models were used to assess the effect of baseline CSF sTREM2 plus sTNFR2 on CSF t-tau, p-tau, and Aβ42 levels (dependent variable) after controlling for well-known covariates of age, sex, education years, and *APOE*ε*4* status. The interaction between CSF sTREM2 and sTNFR2 on the AD biomarkers was also assessed for significance. Effect size was calculated using partial η^2^. F-test for lack-of-fit and residual plots were used to assess the model linearity and fit.

#### Model 3

This analysis was performed only for the ADNI cohort, as the replication memory clinic cohort had a maximum follow-up period of only 15 months. To assess whether the effect of baseline sTNFR2 and sTREM2 on future cognitive decline was dependent or independent of the effect on AD biomarker CSF t-tau, linear mixed-effects regression models were applied to the MCI and AD dementia A+ T+ groups. CDR-SB at each visit was the dependent variable. The fixed main effects for sTREM2, sTNFR2, t-tau, and visit number, as well as the interactions between each biomarker and visit number, were evaluated. A random intercept for each patient was included in all models. The visit number was defined as the follow-up duration of the neuropsychological testing in years (with baseline set at zero). Covariates of age, sex, years of education, *APOE*ε*4* status, and CSF Aβ42 were controlled for in each analysis. In addition, 95% confidence intervals (CIs) and Benjamini-Hochberg adjusted false discovery rate (FDR) *P*-values were calculated. Higher order interactions between sTREM2 and sTNFR2 together on longitudinal clinical outcomes were not assessed given challenges in interpretation. A sensitivity analysis was next conducted with and without the covariates and using p-tau instead of t-tau (given the concern for collinearly with both CSF t-tau and p-tau in the model) to evaluate the reliability of these results. Additionally Model 3 (without sTNFR2) results were corroborated in a sensitivity analysis among a larger number of ADNI participants with sTREM2 data alone (demographics in Supplementary Tables 11, 12).

### Data Availability

The ADNI data analyzed are available in the ADNI repository, http://adni.loni.usc.edu/.

## Results

Demographic details of participants from the ADNI cohort (A+ T+) and the memory clinic replication cohort are presented in Table 1. The above two cohorts differed in age, and *APOE*ε*4* status, in addition to biomarker variables and baseline cognitive scores. Between A+T+ CN, MCI and dementia groups in ADNI, only CSF Aβ levels differed between them (F = 3.91, *p* = 0.023) but not CSF t-tau, p-tau, sTREM2 and sTNFR2 analytes (Supplementary Figure 1).

### ADNI and Replication Memory Clinic Cohorts

Among the MCI and AD dementia A+ T+ participants in the ADNI cohort, univariate analysis demonstrated that sTNFR2 and sTREM2 were significantly correlated with each other and with CSF t-tau and p-tau but not with Aβ42. This significant positive correlation between sTNFR2 and sTREM2 was replicated in the memory clinic participants with MCI, but only the sTNFR2 positive correlation with CSF t-tau and p-tau met the significance threshold (Table 2).

**Table 2:** Pearson correlations between sTNFR2/sTREM2 and Aβ42, t-tau, and p-tau for participants from the Alzheimer's Disease Neuroimaging Initiative (ADNI) and replication memory clinic cohorts (participants meeting A+ T+ criteria, includes those with concomitant sTREM2 and sTNFR2 data).

**Cohort**	**Analyte**	**Log_**2**_ sTREM2**	**Log_**2**_ sTNFR2**	**Log_**2**_ Aβ42**	**Log_**2**_ t-tau**	**Log_**2**_ p-tau**
		**Correlation (*P*-value)**	**Correlation (*P*-value)**	**Correlation (*P*-value)**	**Correlation (*P*-value)**	**Correlation (*P*-value)**
ADNI MCI (*n* = 67)	sTREM2	1	0.67 (<0.0001)^**^	0.23 (0.059)	0.31 (0.01)^**^	0.31 (0.008)^**^
	sTNFR2	0.61 (<0.0001)^**^	1	0.072 (0.56)	0.49 (<0.0001)^**^	0.48 (<0.0001)^**^
ADNI AD dementia (*n* = 42)	sTREM2	1	0.75 (<0.0001)^**^	0.23 (0.13)	0.30 (0.052)	0.24 (0.11)
	sTNFR2	0.75 (<0.0001)^**^	1	0.21 (0.16)	0.31 (0.042)^*^	0.26 (0.088)
Replication memory clinic (*n* = 48)	sTREM2^a^	1	0.31 (0.042)^*^	0.058 (0.71)	0.17 (0.25)	0.19 (0.20)
	sTNFR2	0.31 (0.042)^*^	1	0.27 (0.058)	0.72 (<0.0001)^*^	0.72 (<0.0001)^*^

*AD, Alzheimer's disease; MCI, mild cognitive impairment; sTNFR 2, soluble tumor necrosis factor receptor 2; sTREM2, soluble triggering receptor expressed on myeloid cells 2*.

a *n = 43*.

* *P ≤ 0.05*.

** *P ≤ 0.01 and False Discovery Rate, p = 0.05*.

#### Models 1 and 2

**Figure 1:**
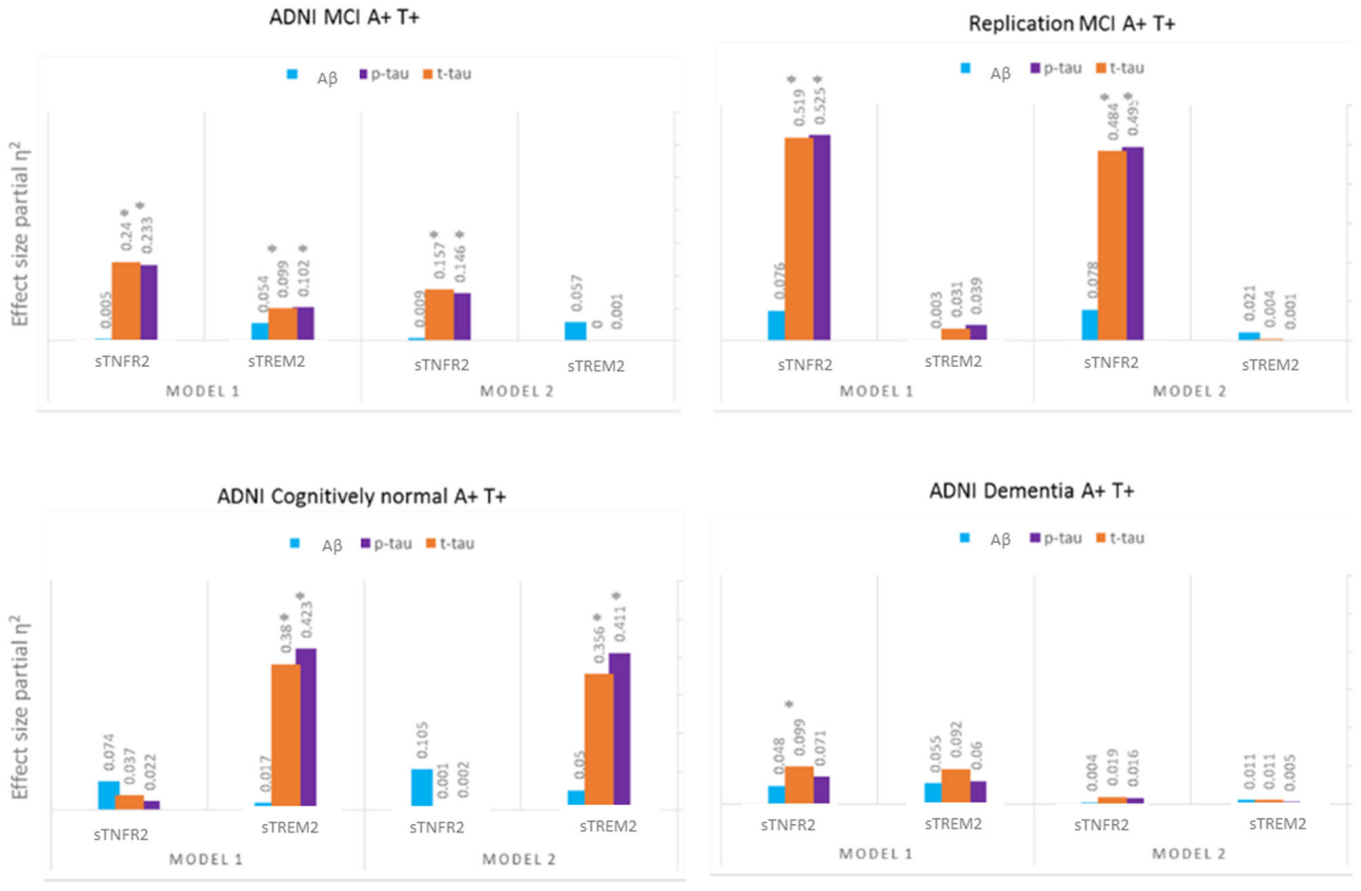
Effect sizes for baseline CSF sTREM2 and sTNFR2 in linear models predicting baseline CSF t-tau, p-tau, and Aβ42 in A+ T+ participants with MCI (ADNI cohort and replication memory clinic cohort) or AD dementia (ADNI cohort) and in A+ T+ cognitively normal participants. *P ≤ 0.05.

For ADNI MCI A+ T+ participants, both sTNFR2 and sTREM2 significantly predicted CSF p-tau and t-tau levels but not Aβ42 level (Model 1). The effect sizes were higher for sTNFR2 than for sTREM2. In Model 2, only sTNFR2 significantly predicted CSF p-tau and t-tau levels (Figure 1, Table 3). For ADNI AD dementia A+ T+ participants, sTNFR2 significantly predicted CSF t-tau levels but not CSF p-tau or Aβ42 levels (Model 1). In Model 2, both analytes failed to meet significance (Figure 1, Table 4). sTREM2, sTNFR2 interactions on AD biomarker outcomes were checked and were found to be non-significant for both MCI and dementia A+ T+ participants and were removed from model fits.

**Table 3:** Key results of the general linear model with CSF Aβ42, t-tau, and p-tau as the dependent variables and covariates: age, sex, education, APOEε4 status, among participants from the Alzheimer's Disease Neuroimaging Initiative cohort with mild cognitive impairment (participants meeting A+ T+ criteria).

**Model 1: Main effects: CSF sTNFR2** ***OR*** **sTREM2**								
**Main effect**	**Dependent variable**	**Type III sum of squares**	**df**	**Mean square**	**F**	***P*-value**	**R^**2**^**	**Partial eta squared**
sTNFR2	Aβ42	0.083	1.65	0.054	0.342	0.561	0.005	0.005
	t-tau	2.395	1.65	2.395	20.538	<0.0001^**^	0.24	0.24
	p-tau	2.858	1.65	2.858	19.711	<0.0001^**^	0.233	0.233
sTREM2	Aβ42	0.553	1.65	0.553	3.698	0.059	0.054	0.054
	t-tau	0.986	1.65	0.986	7.13	0.01^**^	0.099	0.099
	p-tau	1.252	1.65	1.252	7.374	0.008^**^	0.102	0.102
**Model 2: Main effects: CSF sTNFR2** ***AND*** **sTREM2**								
sTNFR2	Aβ42	0.083	1.64	0.083	0.549	0.461	0.062	0.009
	t-tau	1.411	1.64	1.411	11.921	0.001^**^	0.24	0.157
	p-tau	1.616	1.64	1.616	10.985	0.002^**^	0.233	0.146
sTREM2	Aβ42	0.582	1.64	0.582	3.864	0.054	0.062	0.057
	t-tau	0.002	1.64	0.002	0.021	0.886	0.24	0
	p-tau	0.009	1.64	0.009	0.064	0.801	0.233	0.001

*CSF, cerebrospinal fluid; df, degrees of freedom; sTNFR 2, soluble tumor necrosis factor receptor 2; sTREM2, soluble triggering receptor expressed on myeloid cells 2*.

* *P ≤ 0.05*.

** *FDR ≤ 0.05*.

**Table 4:** Key results of the general linear model with CSF Aβ42, t-tau, and p-tau as the dependent variables and covariates: age, sex, education, APOEε4 status, among participants from the Alzheimer's Disease Neuroimaging Initiative cohort with Alzheimer's disease dementia (participants meeting A+ T+ criteria).

**Model 1: Main effects: CSF sTNFR2** ***OR*** **sTREM2**								
**Main effect**	**Dependent variable**	**Type III sum of squares**	**df**	**Mean square**	**F**	***P*-value**	**R^**2**^**	**Partial eta squared**
sTNFR2	Aβ42	0.404	1.40	0.404	2.003	0.165	0.048	0.048
	t-tau	0.65	1.40	0.65	4.408	0.042^*^	0.099	0.099
	p-tau	0.586	1.40	0.586	3.051	0.088	0.071	0.071
sTREM2	Aβ42	0.462	1.40	0.462	2.307	0.137	0.055	0.055
	t-tau	0.599	1.40	0.599	4.029	0.052	0.092	0.092
	p-tau	0.498	1.40	0.498	2.563	0.117	0.06	0.06
**Model 2: Main effects: CSF sTNFR2** ***AND*** **sTREM2**								
sTNFR2	Aβ42	0.035	1.39	0.035	0.17	0.682	0.059	0.004
	t-tau	0.114	1.39	0.114	0.763	0.388	0.109	0.019
	p-tau	0.126	1.39	0.126	0.642	0.428	0.075	0.016
sTREM2	Aβ42	0.093	1.39	0.093	0.453	0.505	0.059	0.011
	t-tau	0.063	1.39	0.063	0.424	0.519	1.09	0.011
	p-tau	0.038	1.39	0.038	0.193	0.663	0.075	0.005

*CSF, cerebrospinal fluid; df, degrees of freedom; sTNFR 2, soluble tumor necrosis factor receptor 2; sTREM2, soluble triggering receptor expressed on myeloid cells 2*.

* *P ≤ 0.05*.

** *FDR ≤ 0.05*.

Among participants in the replication memory clinic cohort, sTNFR2 (but not sTREM2) significantly predicted CSF p-tau and t-tau levels but not CSF Aβ42 level (Model 1). In Model 2, only sTNFR2 significantly predicted CSF p-tau and t-tau levels (Figure 1, Table 5). sTREM2, sTNFR2 interactions on AD biomarker outcomes in the replication cohort were again non-significant.

**Table 5:** Key results of the general linear model with CSF Aβ42, t-tau, and p-tau as the dependent variables and covariates: age, sex, education, APOEε4 status, among mild cognitive impairment participants from a replication memory clinic cohort (participants meeting A+ T+ criteria).

**Main effect**	**Dependent variable**	**Type III sum of squares**	**df**	**Mean square**	**F**	***P*-value**	**R^**2**^**	**Partial eta squared**
**Model 1: Main effects: CSF sTNFR2** ***OR*** **sTREM2**								
sTNFR2	Aβ42	1.093	1.46	1.093	3.775	0.058	0.076	0.076
	t-tau	20.951	1.46	20.951	49.543	<0.0001^**^	0.519	0.519
	p-tau	11.073	1.46	11.073	50.789	<0.0001^**^	0.525	0.525
sTREM2	Aβ42	0.035	1.41	0.035	0.139	0.711	0.003	0.003
	t-tau	1.061	1.41	1.061	1.315	0.258	0.031	0.031
	p-tau	0.747	1.41	0.747	1.685	0.202	0.039	0.039
**Model 2: Main effects: CSF sTNFR2** ***AND*** **sTREM2**								
sTNFR2	Aβ42	0.807	1.40	0.807	3.367	0.074	0.081	0.078
	t-tau	16.004	1.40	16.004	37.482	<0.0001^**^	0.5	0.484
	p-tau	9.006	1.40	9.006	39.252	<0.0001^**^	0.515	0.495
sTREM2	Aβ42	0.21	1.40	0.21	0.875	0.355	0.081	0.021
	t-tau	0.071	1.40	0.071	0.167	0.685	0.5	0.004
	p-tau	0.013	1.40	0.013	0.056	0.815	0.515	0.001

*CSF, cerebrospinal fluid; df, degrees of freedom; sTNFR 2, soluble tumor necrosis factor receptor 2; sTREM2, soluble triggering receptor expressed on myeloid cells 2*.

* *P ≤ 0.05*.

** *FDR ≤ 0.05*.

#### Model 3

**Figure 2:**
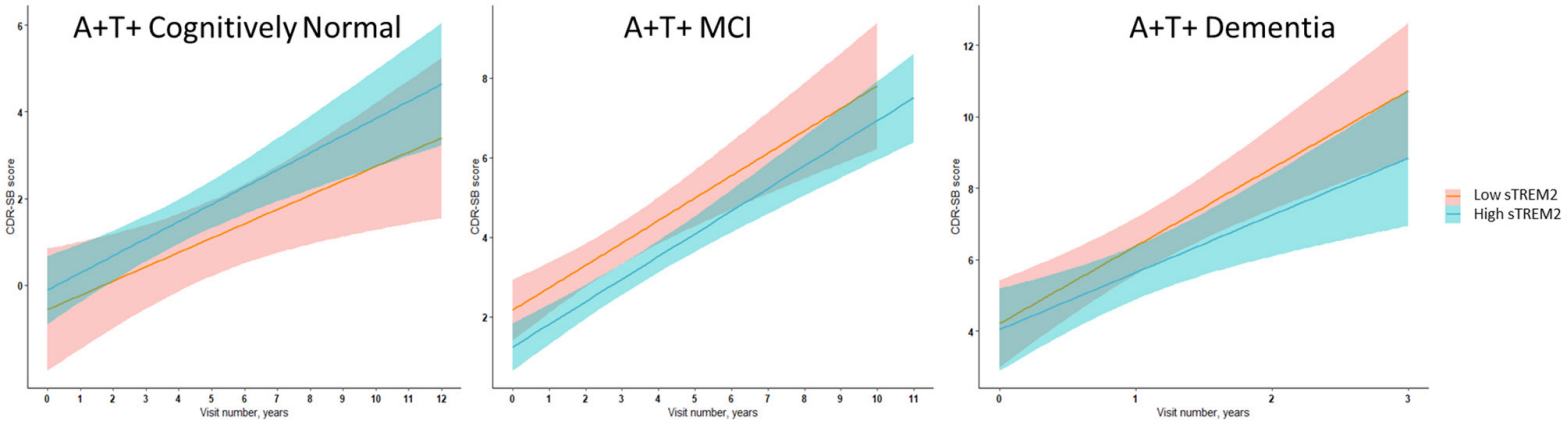
Illustrative linear regression with 95% CI plots for sTREM2 levels at the 25th and 75th percentiles at baseline vs. CDR-SB score longitudinally for A+ T+ participants with MCI stage of AD (*n* = 111) or AD dementia (*n* = 57) and in A+ T+ cognitively normal participants (*n* = 16) also used for sensitivity analyses described in Supplementary Tables 11–15.

In ADNI MCI A+ T+ participants, neither CSF sTREM2 nor sTNFR2 levels predicted future cognitive decline on CDR-SB when taking into account CSF p-tau or t-tau levels (Figure 2, Table 6). In a sensitivity analysis with larger number of participants with sTREM2 and AD biomarkers alone, the above results were again corroborated (Supplementary Table 13).

**Table 6:** Key results of the linear mixed-effects regression model with CDR-SB as the dependent variable among participants with A+ T+ mild cognitive impairment from the Alzheimer's Disease Neuroimaging Initiative cohort (Model 3).

**Parameter**	**Estimate**	**Standard error**	**df**	**t**	***P*-value**	**95% confidence interval**	
						**Lower bound**	**Upper bound**
Intercept	3.991006	13.69949	107.56	0.291	0.771	−23.165	31.14702
sTREM2	−0.42004	0.632681	107.895	−0.664	0.508	−1.67413	0.834061
sTNFR2	0.829879	3.489585	109.082	0.238	0.812	−6.08631	7.746066
t-tau	0.313797	1.12039	108.769	0.28	0.78	−1.90683	2.534427
Visit number in years	2.761182	2.621731	348.072	1.053	0.293	−2.39525	7.917609
Visit number in years × sTREM2	0.015941	0.120067	360.652	0.133	0.894	−0.22018	0.25206
Visit number in years × sTNFR2	0.129465	0.720227	362.272	0.18	0.857	−1.28689	1.545817
Visit number in years × t-tau	−0.22548	0.238439	365.207	−0.946	0.345	−0.69436	0.243411

*CSF sTREM2 × visit number + CSF sTNFR2 × visit number, CSF t-tau × visit number + CSF sTREM2 + CSF sTNFR2 + CSF t-tau + visit number (fixed effect). CDR-SB, Clinical Dementia Rating–Sum of Boxes; CSF, cerebrospinal fluid; df, degrees of freedom; sTNFR 2, soluble tumor necrosis factor receptor 2; sTREM2, soluble triggering receptor expressed on myeloid cells 2*.

In ADNI AD dementia A+ T+ participants, higher CSF sTREM2 predicted lower future cognitive decline on CDR-SB independent of CSF t-tau levels. With every doubling of CSF sTREM2 levels, the longitudinal change in CDR-SB decreased by 1.3 points (β = −1.33, df = 115.2, t = −2.27, *P* = 0.006, FDR = 0.048) (Figure 2, Table 7). In a sensitivity analysis using p-tau instead of t-tau, the same directional trend was observed (β = −1.28, df = 114.7, t = −2.69, *P* = 0.008, FDR = 0.064). On further evaluating the robustness of this effect among a larger number of participants with sTREM2 and AD biomarkers alone, the significance and directionality of the results were again corroborated (Supplementary Table 14).

**Table 7:** Key results of the linear mixed-effects regression model with CDR-SB as the dependent variable among participants with A+ T+ AD dementia from the Alzheimer's Disease Neuroimaging Initiative (ADNI) cohort (Model 3).

**Parameter**	**Estimate**	**Standard error**	**df**	**t**	***P*-value**	**95% confidence interval**		**FDR**
						**Lower bound**	**Upper bound**	
Intercept	5.76527	14.38224	62.274	0.401	0.69	−22.9819	34.51244	1
sTREM2	0.096417	0.961389	61.919	0.1	0.92	−1.82542	2.018256	0.92
sTNFR2	1.417211	4.269298	61.595	0.332	0.741	−7.11811	9.952532	0.988
t-tau	−0.31314	1.091746	62.298	−0.287	0.775	−2.4953	1.869025	0.885
Visit number in years	9.80077	7.13377	113.013	1.374	0.172	−4.3325	23.93404	0.458
Visit number in years × sTREM2	−1.33428	0.47665	115.165	−2.799	0.006^*^	−2.27841	−0.39014	0.048^*^
Visit number in years × sTNFR2	2.457261	2.061383	116.28	1.192	0.236	−1.62547	6.539987	0.472
Visit number in years × t-tau	0.983455	0.56413	113.405	1.743	0.084	−0.13415	2.101055	0.336

*CSF sTREM2 × visit number + CSF sTNFR2 × visit number, CSF t-tau × visit number + CSF sTREM2 + CSF sTNFR2 + CSF t-tau + visit number (fixed effect). CDR-SB, Clinical Dementia Rating–Sum of Boxes; CSF, cerebrospinal fluid; df, degrees of freedom; FDR, false discovery rate; sTNFR 2, soluble tumor necrosis factor receptor 2; sTREM2, soluble triggering receptor expressed on myeloid cells 2*.

* *P ≤ 0.05*.

### Exploratory Analysis in ADNI CN Participants

For all ADNI CN participants, all A and T groups combined, sTNFR2 and sTREM2 were correlated (ρ = 0.487, *p* < 0.0001). In the A– T– subgroup, sTNFR2 and sTREM2 were modestly correlated with each other (ρ = 0.35, *p* = 0.036) and each analyte was significantly related to CSF t-tau (sTNFR2 ρ = 0.38, *p* = 0.023 and sTREM2 ρ = 0.35, *p* = 0.034). In the A+ T+, A+ T–, and A– T+ subgroups, sTNFR2 and sTREM2 were not significantly correlated with each other. Only sTREM2 was correlated with CSF t-tau and p-tau in the A+ T+ subgroup, whereas only sTNFR2 was significantly correlated with CSF t-tau and p-tau in the A- T+ subgroup (Supplementary Table 2).

#### Models 1 and 2

**Figure 3:**
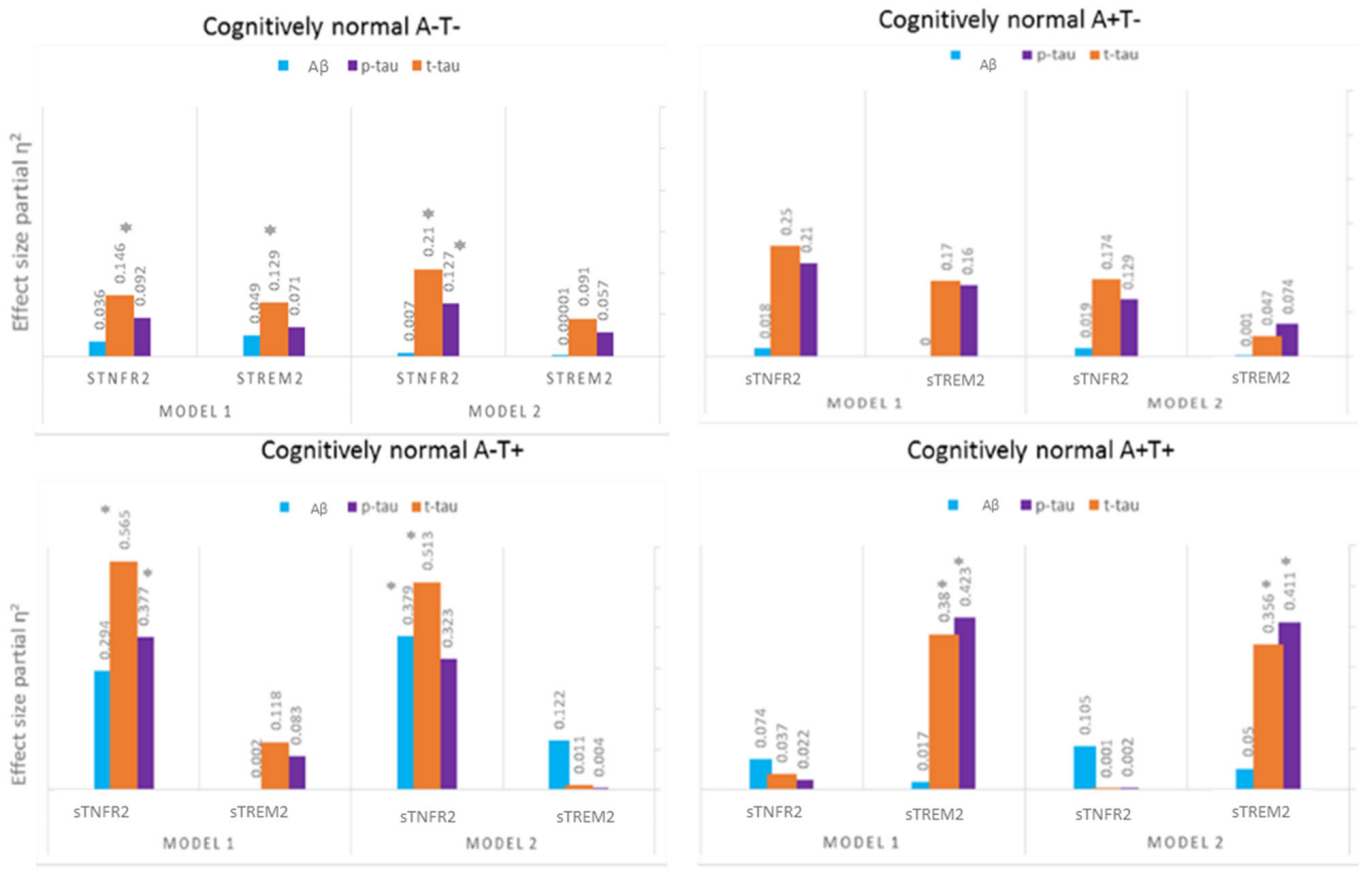
Effect sizes for baseline CSF sTREM2 and TNFR2 in linear models predicting baseline CSF t-tau, p-tau, and Aβ42 among A– T–, A– T+, A+ T–, and A+ T+ cognitively normal participants. *P ≤ 0.05.

Among A+ T+ CN participants, only sTREM2 predicted CSF t-tau and p-tau levels but not CSF Aβ42 level (Model 1). In Model 2, sTREM2 again significantly predicted CSF p-tau and t-tau levels (Figure 3, Supplementary Table 3). sTREM2 and sTNFR2 interaction on AD biomarker outcomes among A+ T+ CN participants were again non-significant.

Among A+ T– CN participants, neither analyte significantly predicted CSF p-tau and t-tau levels (Figure 3, Supplementary Table 4).

Among A– T+ CN participants, sTNFR2 predicted t-tau and p-tau levels (Model 1). In Model 2, sTNFR2 significantly predicted CSF t-tau and Aβ42 levels (Figure 3, Supplementary Table 5).

Among A– T– CN participants alone, the interaction of sTNFR2 and sTREM2 was significant on t-tau and p-tau levels (Model 1) (Supplementary Table 6).

#### Model 3

Among all CN participants combined, neither sTNFR2 nor sTREM2 predicted longitudinal cognitive change after accounting for CSF AD biomarkers (data not presented).

Among A+ T+ CN participants, higher sTREM2 levels predicted more longitudinal cognitive change after accounting for CSF t-tau but this result was not significant after FDR correction (β = 0.94, df = 21.94, t = 2.43, *P* = 0.023, FDR = 0.092) (Supplementary Figure 1, Supplementary Table 7). In a sensitivity analysis with a slightly larger number of participants with sTREM2 and AD biomarkers alone, the above results were again corroborated and now met FDR threshold (Supplementary Table 15).

Among A– T+, A+ T– and A– T– CN participants, neither sTNFR2 nor sTREM2 predicted longitudinal cognitive change after accounting for CSF AD biomarkers after FDR correction (Supplementary Tables 8–10).

## Discussion

This study demonstrates that the CSF levels of sTREM2 and sTNFR2 are dynamic in relation to p-tau and t-tau biomarkers over the temporal stages of AD. A positive correlation was seen between CSF levels of sTREM2 and sTNFR2 in A+ T+ participants with AD dementia (ADNI) or MCI (both cohorts). Among the above participants, sTNFR2 rather than sTREM2 explained most of the variance in relation to CSF t-tau and p-tau, whereas sTREM2 rather than sTNFR2 explained most of the variance in the same biomarkers among A+ T+ CN individuals (ADNI). These results are consistent with previous reports that demonstrated a positive correlation between CSF sTREM2 and t-tau and p-tau in participants with MCI (Heslegrave et al., 2016; Piccio et al., 2016; Suárez-Calvet et al., 2016a,b, 2019), and among preclinical AD with low CSF Aβ_42_ and high total-tau or p-tau levels (Ma et al., 2020) but our results extend previous reports by demonstrating the limited significance of CSF sTREM2 in explaining CSF t-tau and p-tau variance when taking CSF sTNFR2 levels into account among participants in various AD clinical stages (with the exception of A+ T+ CN). There was no significant interaction between sTNFR2 and sTREM2 levels on AD biomarkers in A+T+ CN, MCI and dementia groups but this interaction was significant in the A− T− CN group.

Prior analysis in the ADNI cohort had noted that higher CSF sTREM2 levels at baseline were associated with slower rates of Aβ accumulation as assessed by amyloid PET over 2 years, with the largest effect in the MCI and dementia stages of AD (Ewers et al., 2020). Consistent with this, we note that the effect of CSF sTREM2 on cognitive decline (i.e., higher level of CSF sTREM2 relates to slower longitudinal decline on CDR-SB scores) was significant at the A+ T+ AD dementia stage, possibly subsequent to its effect on Aβ accumulation that appears maximal at the MCI stage and this interestingly was independent of its association with CSF t-tau and p-tau levels. Additionally, we found that sTREM2 levels explain less of the variance in CSF Aβ42 similar to previous reports (Suárez-Calvet et al., 2016a,b, 2019; Ma et al., 2020).

In contrast, CSF sTNFR2 levels were associated with CSF t-tau and p-tau levels in the A+T+ MCI and dementia stages but did not predict rates of cognitive decline on CDR-SB on longitudinal follow up over subsequent years. However, among A+ T+ MCI or AD dementia groups in ADNI, we had previously reported that the interaction between *TNFRSF1B* gene variants and CSF sTNFR2 levels relates to CSF t-tau and p-tau levels and longitudinal cognitive change over 1 year (Pillai et al., 2021). This suggests that the sTNFR2 levels are impacted by both CSF t-tau and p-tau levels and *TNFRSF1B* gene variant status, and mitigate cognitive decline over the short term but do not significantly impact cognitive outcomes over the longer term as the disease continues to evolve.

Our exploratory analysis among CN participants further notes that the relative association of CSF sTREM2 on markers of neuronal injury and neurofibrillary tangles (CSF t-tau and p-tau) is higher in the preclinical stage of AD (A+ T+ CN) than in the A+ T+ MCI or dementia stages. Consistent with this, higher CSF sTREM2 levels that relate to higher CSF t-tau and p-tau levels at the preclinical AD stage was associated with greater cognitive decline, unlike in the dementia stage, in which higher CSF sTREM2 levels were associated with slower cognitive decline (Figure 2). These results among CN groups which were limited to CSF measures of Aβ and tau are preliminary given the smaller number of subjects analyzed with both CSF sTREM2 and sTNFR2 data. Although higher levels of CSF sTREM2 do not necessarily relate to cognitive outcomes favorably in the preclinical stage with concurrent increase in CSF t-tau and p-tau in our data, they are still a marker of slower rate of clinical progression longitudinally in later disease stages. This is also supported by previous reports (Ewers et al., 2019; Franzmeier et al., 2020).

Taken together our results suggest that there is a temporal window that should be considered for future therapeutic options targeting sTREM2- and sTNFR2-related inflammatory pathways and for the use of CSF sTREM2 in combination with other CSF AD and inflammatory biomarkers in evaluating its clinical significance. The temporal changes in CSF sTREM2 across different AD stages in this study parallels what has been described previously (Suárez-Calvet et al., 2016b, 2019). Based on prior models on the temporal evolution of AD biomarkers (Jack and Holtzman, 2013), Figure 4 summarizes a hypothetical model based on the key findings of CSF sTREM2- and sTNFR2 relationships to Aβ and tau levels in the different stages of AD, plotting biomarker severity (degree of abnormality) vs. disease stage. The key insight here being that the time course of change among the distinct inflammation related markers (sTREM2, sTNFR2) with the progression of AD stages are very different.

**Figure 4:**
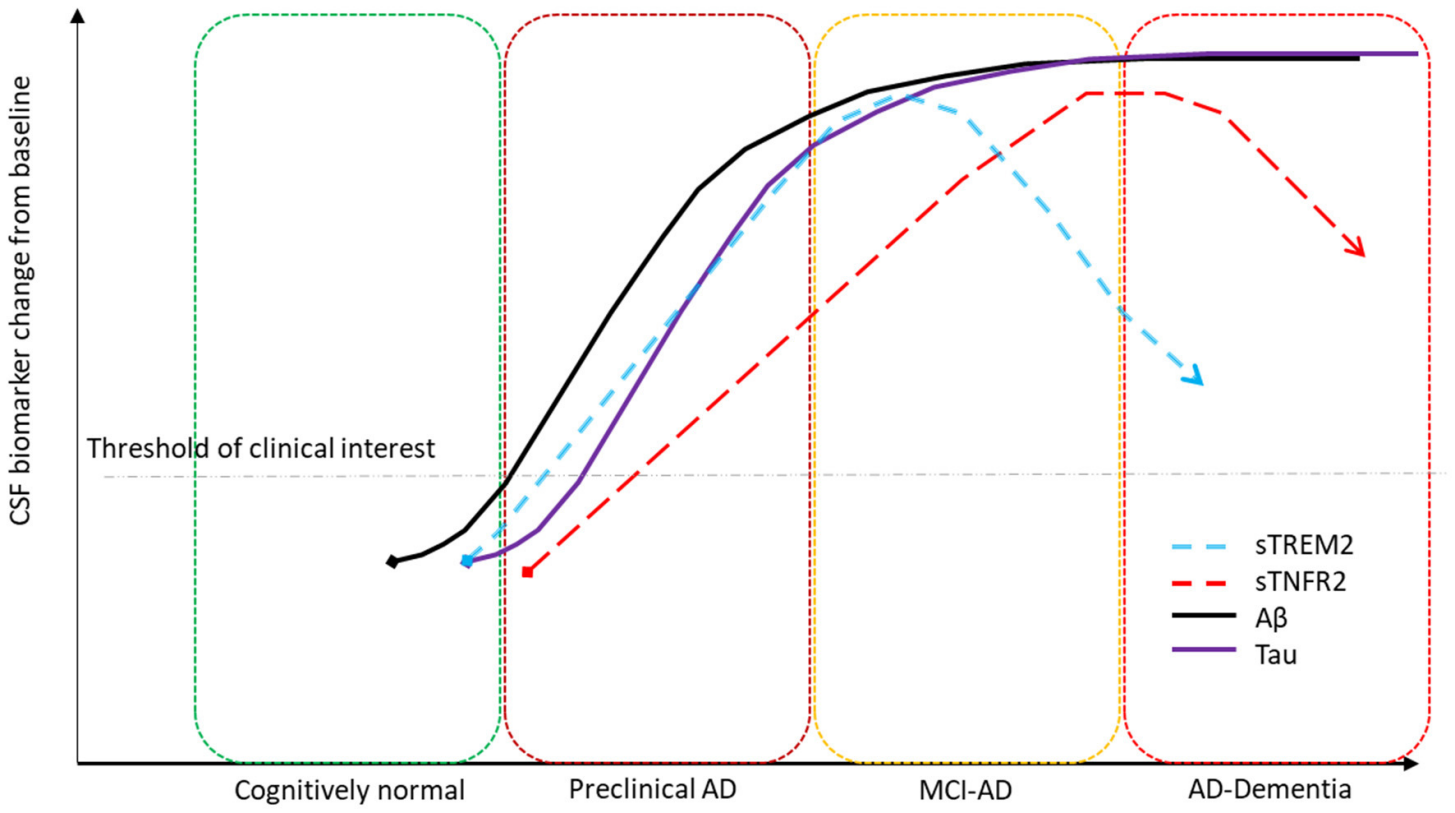
Hypothetical model summarizing key biomarker findings. CSF sTREM2 significantly correlates to CSF p-tau levels in the A+ T+ preclinical and MCI stages of AD and CSF sTNFR2 significantly correlates to CSF p-tau levels in the A+ T+ MCI and dementia stages of AD.

Analysis within the CN subgroups was exploratory given the small number of participants. Nevertheless, we could still delineate some patterns of interest with respect to sTREM2 that needs future validation. First, preclinical AD (CN A+ T+) appears to be a transition state in which the relationship noted between sTREM2, sTNFR2 and CSF t-tau or p-tau in all other stages of AD appears to be reversed, suggesting that in this stage, sTREM2 explains more of the variance of CSF t-tau and p-tau than does sTNFR2. This is consistent with prior results among preclinical AD with low Aβ_42_ and high total-tau or p-tau in the CABLE study (Ma et al., 2020). Within the CN A+ T+ group, higher sTREM2 levels was associated with more cognitive decline (unlike in AD dementia). This finding is consistent with results from the DIAN Study, in which an increase in CSF sTREM2 among autosomal dominant mutation carriers was found to differ from the level in normal controls 5 years before symptom onset, with this increase followed by Aβ, tau, and associated neurodegenerative changes; in advanced stages of the disease in the DIAN cohort, this difference did not reach statistical significance (Suárez-Calvet et al., 2016c). A second pattern we observed in the current study was that higher levels of sTREM2 appear to be related to slower cognitive decline among AD stages in which sTREM2 is not strongly correlated with neurodegeneration markers (i.e., AD dementia), but this was not the case among AD stages that demonstrated a strong correlation between sTREM2 and neurodegeneration markers (CN A+ T+). This suggests that CSF levels of sTREM2 alone are likely not good independent prognosticators of future clinical decline but could reflect a more complex interplay of immune cell activation in relation to neurodegenerative changes in different AD stages.

The statistical interaction noted between sTNFR2 and sTREM2 among CN A-T- participants shows higher levels of both sTNFR2 and sTREM2 together impact t-tau and p-tau levels more than we would expect if they acted independently, this appears to parallel the reports from animal studies where TNFR2 activation levels were related to transcriptional regulation of TREM2 (Gao et al., 2017). It is possible that the lack of statistical interaction between sTNFR2 and sTREM2 levels on AD biomarkers among A+T+ CN, MCI and dementia suggests altered TNFR2 and TREM2 pathway activations in AD. As the current study is an association study of biomarkers, future studies in AD models are needed to evaluate the mechanistic relationship between TNFR2 and TREM2 in AD.

## Limitations

Biomarker changes alone may not reflect completely the pathophysiological roles of TNFR2 and TREM2 at different AD stages and these should not be interpreted as providing mechanistic insights. Further, all models evaluating CSF sTNFR2 and CSF sTREM2s impact on AD biomarkers relied on crossectional data. Crossectional data do not provide a window into within subject temporal trajectories of CSF sTNFR2 and sTREM2s changes, therefore future longitudinal evaluations of these inflammatory analytes are needed. These study results are most robust for the A+ T+ MCI and dementia stages of AD given the relatively large number of participants and the replication of results across two different A+ T+ MCI cohorts. However, the results in the A+ T+ CN group was consistent with reports from prior studies and therefore provide confidence to our results. Given the limited follow-up (15 months) in the replication cohort, the findings from this group could not be used to corroborate the effects of these analytes longitudinally from ADNI. These findings should also be corroborated in a cohort with different recruitment goals than ADNI, as the ADNI cohort is predominantly White with a high education attainment. We did not screen participants for possible TREM2 mutations, as the likelihood of these mutations is low (Suárez-Calvet et al., 2019). Additionally, lack of neuropathologic confirmation also limits our understanding of the role of mixed pathology.

There were also known differences between the ADNI and the replication cohorts. The replication cohort included a sample of memory clinic participants with a faster rate of disease progression than participants in the ADNI cohort (Pillai et al., 2020). Additionally, the replication cohort also included a few atypical AD participants with highly elevated CSF t-tau levels (Pillai et al., 2019a) and had a higher frequency of *APOE* ε4 carriers, unlike typical amnestic MCI participants in the ADNI cohort. The positive correlation between sTNFR2 and CSF t-tau and p-tau levels was still consistent within each cohort, but the replication cohort had higher mean t-tau and p-tau levels than the ADNI cohort even when the CSF levels were compared using the same measurement technique as previously reported (Pillai et al., 2020). Mean sTNFR2 levels correlating with neurodegeneration biomarkers were therefore much higher in the A+ T+ MCI replication cohort than in the A+ T+ MCI ADNI cohort.

The differences between the ADNI A+ T+ MCI and the replication cohort A+ T+ MCI also gives us pause in extrapolating the current results to future validation cohorts with different AD clinical and pathological characteristics given the heterogeneity in clinical, biomarker and neuropathology phenotypes of AD (Murray et al., 2011; Pillai et al., 2019a; Suárez-Calvet et al., 2019). The relationship between sTREM2 and CSF t-tau was also stronger in the dementia stage in a prior report by our group (Bekris et al., 2018). Perhaps these differences reflect the variability in clinical symptoms even within the broad stage of AD dementia between cohorts. It is also likely that *APOE* ε4 carrier rates can vary from study to study impacting longitudinal results (Tsuang et al., 1996; Franzmeier et al., 2020). Presence of mixed pathology and the differences in recruitment biases to atypical AD should also be considered when comparisons are made between cohorts in longitudinal outcomes (Dubois et al., 2007; Pillai et al., 2016). It is likely these are some possible reasons behind significant differences in sTREM2 levels reported by prior studies across the AD spectrum (Kleinberger et al., 2014; Gispert et al., 2016b; Henjum et al., 2016; Bekris et al., 2018; Suárez-Calvet et al., 2019; Knapskog et al., 2020).

Type II errors also must be considered in this study given the smaller number of participants within some CN subgroups, as smaller effect sizes could have been missed. It is possible that with a larger sample size among the CN subgroups, some variables of significance could become more salient in the consideration of cognitive and AD biomarker outcomes. Independent replication in larger cohorts using the same biomarkers as those used in ADNI would allow us to clarify this point.

## Conclusions

Our results suggest that the levels of both sTREM2 and sTNFR2 vary dynamically in relation to neurodegenerative biomarkers at different AD stages. This implies that the utility of distinct inflammation related biomarkers in tracking AD temporal progression and their role in predicting clinical outcomes are also expected to differ based on disease stage.

## Data Availability Statement

The datasets presented in this study can be found in online repositories. The names of the repository/repositories and accession number(s) can be found at: http://adni.loni.usc.edu/.

## Ethics Statement

The studies involving human participants were reviewed and approved by Cleveland Clinic. The patients/participants provided their written informed consent to participate in this study.

## Author Contributions

JP obtained funding, design and conceptualized study, analyzed the data, interpreted the data, and drafted the manuscript for intellectual content. MK analyzed the data and revised the manuscript for intellectual content. JB design of study, interpreted the data, and revised the manuscript for intellectual content. JL interpreted the data and revised the manuscript for intellectual content. LB organized the data, interpreted the data, and revised the manuscript for intellectual content. All authors read and approved the final manuscript.

## Conflict of Interest

JP received research funding from the National Institutes of Health, Alzheimer's Association, and Keep Memory Alive Foundation. JL has received consulting fees from Acadia, Aptnyx, Biogen, Eisai, GE Healthcare, Sanofi, and Takeda and grant support from the Alzheimer's Association, Alzheimer's Drug Discovery Foundation, Biogen, Department of Defense, GE Healthcare, Genzyme/Sanofi, Lewy Body Dementia Association, Michael J. Fox Foundation, and National Institute of Health NIA, NINDS. The remaining authors declare that the research was conducted in the absence of any commercial or financial relationships that could be construed as a potential conflict of interest.

## Footnotes

^1^Biomarkers Consortium Project. Use of Targeted Multiplex Proteomic Strategies to Identify Novel Cerebrospinal Fluid (CSF) Biomarkers in Alzheimer's Disease (AD). Data Primer. Version 28 Dec 2011.

## Supplementary Material

Supplementary Figure 1 Box plot for CSF levels of log_2_ sTREM2, log_2_ sTNFR2, log_2_ Abeta, log_2_ total tau within A+ T+ CN, MCI, and dementia groups. The groups differed only on the mean CSF log_2_ Abeta levels (F = 3.91, *p* = 0.023) and not on the other analytes.

Supplementary Table 1 Demographics of cognitively normal participants (includes those with concomitant sTREM2 and sTNFR2 data) in the Alzheimer's Disease Neuroimaging Initiative cohort.

Supplementary Table 2 Pearson correlations between sTNFR2/sTREM2 and Aβ42, t-tau, and p-tau for cognitively normal participants from the Alzheimer's Disease Neuroimaging Initiative cohort.

Supplementary Table 3 Key results of the general linear model with CSF Aβ42, t-tau, and p-tau as the dependent variables among cognitively normal A+ T+ participants from the Alzheimer's Disease Neuroimaging Initiative cohort.

Supplementary Table 4 Key results of the general linear model with CSF Aβ42, t-tau, and p-tau as the dependent variables among cognitively normal A+ T– participants from the Alzheimer's Disease Neuroimaging Initiative cohort.

Supplementary Table 5 Key results of the general linear model results with CSF Aβ42, t-tau, and p-tau as the dependent variables among cognitively normal A– T+ participants from the Alzheimer's Disease Neuroimaging Initiative cohort.

Supplementary Table 6 Key results of the general linear model results with CSF Aβ42, t-tau, and p-tau as the dependent variables among cognitively normal A– T– participants from the Alzheimer's Disease Neuroimaging Initiative cohort.

Supplementary Table 7 Key results of the linear mixed-effects regression model with CDR-SB as the dependent variable among A+ T+ cognitively normal participants from the Alzheimer's Disease Neuroimaging Initiative cohort (Model 3).

Supplementary Table 8 Key results of the linear mixed-effects regression model with CDR-SB as the dependent variable among CN A– T+ cognitively normal participants from the Alzheimer's Disease Neuroimaging Initiative cohort (Model 3).

Supplementary Table 9 Key results of the linear mixed-effects regression model with CDR-SB as the dependent variable among A+ T– cognitively normal participants from the Alzheimer's Disease Neuroimaging Initiative cohort (Model 3).

Supplementary Table 10 Key results of the linear mixed-effects regression model with CDR-SB as the dependent variable among A– T– cognitively normal participants from the Alzheimer's Disease Neuroimaging Initiative cohort (Model 3).

Supplementary Table 11 Demographics of A+T+ participants with CSF sTREM2 values (includes those without concomitant sTNFR2 data) along with CSF AD biomarkers in the Alzheimer's Disease Neuroimaging Initiative cohort for sensitivity analysis.

Supplementary Table 12 Demographics of cognitively normal participants with CSF sTREM2 values (includes those without concomitant sTNFR2 data) along with CSF AD biomarkers in the Alzheimer's Disease Neuroimaging Initiative cohort for sensitivity analysis.

Supplementary Table 13 Sensitivity analysis of linear mixed-effects regression model with CDR-SB as the dependent variable among participants with A+ T+ mild cognitive impairment from the Alzheimer's Disease Neuroimaging Initiative cohort with *n* = 111 with sTREM data alone.

Supplementary Table 14 Sensitivity analysis of linear mixed-effects regression model with CDR-SB as the dependent variable among participants with A+ T+ dementia from the Alzheimer's Disease Neuroimaging Initiative cohort with *n* = 57 with sTREM data alone.

Supplementary Table 15 Sensitivity analysis of linear mixed-effects regression model with CDR-SB as the dependent variable among participants with A+ T+ CN from the Alzheimer's Disease Neuroimaging Initiative cohort with *n* = 16 with sTREM data alone.
