# Supplementary figures and images for: Temporal Ordering of Inflammatory Analytes sTNFR2 and sTREM2 in Relation to Alzheimer's Disease Biomarkers and Clinical Outcomes
Source: Front Aging Neurosci. 2021 Jun 29;13:676744. doi: 10.3389/fnagi.2021.676744 (PMC8279003; doi:10.3389/fnagi.2021.676744)

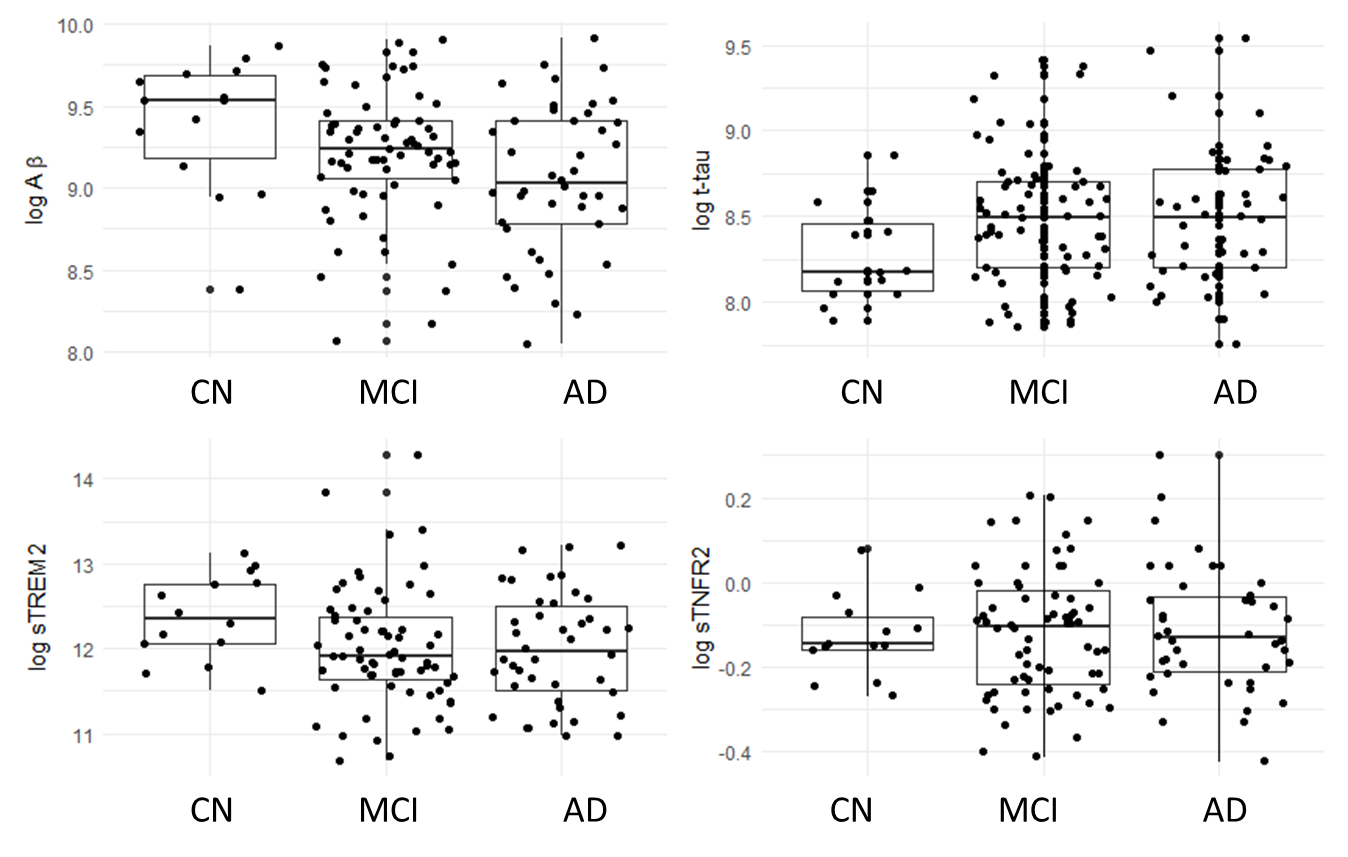

Supplement: Supplementary Figure 1 — Box plot for CSF levels of log2 sTREM2, log2 sTNFR2, log2 Abeta, log2 total tau within A+ T+ CN, MCI, and dementia groups. The groups differed only on the mean CSF log2 Abeta levels (F = 3.91, p = 0.023) and not on the other analytes. [file Image_1.TIF]
