# Supplementary material for: Temporal Ordering of Inflammatory Analytes sTNFR2 and sTREM2 in Relation to Alzheimer's Disease Biomarkers and Clinical Outcomes
Source: Front Aging Neurosci. 2021 Jun 29;13:676744. doi: 10.3389/fnagi.2021.676744 (PMC8279003; doi:10.3389/fnagi.2021.676744)
Supplement: Supplementary Table 1 — Demographics of cognitively normal participants (includes those with concomitant sTREM2 and sTNFR2 data) in the Alzheimer's Disease Neuroimaging Initiative cohort. [file Table_1.DOCX]

**Supplementary Table 1.** Demographics of cognitively normal participants (includes those with concomitant sTREM2 and sTNFR2 data) in the Alzheimer’s Disease Neuroimaging Initiative cohort

| Demographic variable | A– T– (*n* = 35) | A+ T+ (*n* = 14) | A+ T– (*n* = 11) | A– T+ (*n* = 12) |
| --- | --- | --- | --- | --- |
|  | Mean (SD) | Mean (SD) | Mean (SD) | Mean (SD) |
| Age, y | 74.94 (4.61) | 78.72 (5.4) | 73.57 (6.08) | 77.06 (5.06) |
| Sex (% female) | 40% | 42.9% | 36.4% | 66.7% |
| *APOEε4* (%) | 11.4% | 57.1% | 45.5% | 8.3% |
| Patient education, y | 15.09 (2.94) | 17.14 (2.74) | 15.27 (4.3) | 16.42 (2.5) |
| Baseline MMSE score | 28.89 (1.10) | 29.21 (1.05) | 28.55 (1.12) | 29.58 (0.51) |
| Baseline CDR-SB score | 0.014 (0.08) | 0.000 (0) | 0.045 (0.15) | 0.04 (0.14) |
| Log_2_ Aβ42 | 10.47 (0.21) | 9.39 (0.41) | 9.5 (0.38) | 10.51 (0.31) |
| Log_2_ t-tau | 7.58 (0.26) | 8.27 (0.28) | 7.43 (0.33) | 8.26 (0.23) |
| Log_2_ p-tau | 4.08 (0.25) | 4.96 (0.33) | 3.97 (0.35) | 4.78 (0.24) |
| Log_2_ sTREM2 | 11.93 (0.77) | 12.37 (0.51) | 11.39 (0.58) | 12.40 (0.96) |
| Log_2_ sTNFR2 | –0.17 (0.15) | –0.12 (0.094) | –0.27 (0.10) | –0.04 (0.13) |

CDR-SB, Clinical Dementia Rating–Sum of Boxes; MMSE, Mini-Mental State Exam; SD, standard deviation; sTNFR 2, soluble tumor necrosis factor receptor 2; sTREM2, soluble triggering receptor expressed on myeloid cells 2.

**Supplementary Table 2.** Pearson correlations between sTNFR2/sTREM2 and Aβ42, t-tau, and p-tau for cognitively normal participants from the Alzheimer’s Disease Neuroimaging Initiative cohort

| Subgroup | Analyte | Log_2_ sTREM2 | Log_2_ sTNFR2 | Log_2_ Aβ42 | Log_2_ t-tau | Log_2_ p-tau |
| --- | --- | --- | --- | --- | --- | --- |
|  |  | Correlation (*P* value) | Correlation (*P* value) | Correlation (*P* value) | Correlation (*P* value) | Correlation (*P* value) |
| A+ T+  (*n* = 14) | sTREM2 | 1 | 0.27 (0.34) | 0.13 (0.65) | 0.61 (0.019)* | 0.65 (0.012)* |
|  | sTNFR2 | 0.27 (0.34) | 1 | 0.27 (0.34) | 0.19 (0.51) | 0.15 (0.61) |
| A+ T–  (*n* = 11) | sTREM2 | 1 | 0.39 (0.23) | 0.019 (0.96) | 0.37 (0.26) | 0.40 (0.22) |
|  | sTNFR2 | 0.39 (0.23) | 1 | 0.13 (0.69) | 0.50 (0.17) | 0.46 (0.15) |
| A– T+  (*n* = 12) | sTREM2 | 1 | 0.53 (0.074) | 0.042 (0.90) | 0.34 (0.28) | 0.29 (0.36) |
|  | sTNFR2 | 0.53 (0.074) | 1 | 0.54 (0.068) | 0.75 (0.005)** | 0.61 (0.034)* |
| A– T–  (*n* = 35) | sTREM2 | 1 | 0.35 (0.036)* | 0.19 (0.27) | 0.38 (0.023)* | 0.30 (0.076) |
|  | sTNFR2 | 0.35 (0.036)* | 1 | 0.22 (0.20) | 0.35 (0.034)* | 0.26 (0.12) |
| All  (*n* = 73) | sTREM2 | 1 | 0.487 (<0.0001)** | 0.077 (0.52) | 0.511 (<0.0001)** | 0.473 (<0.0001)** |
|  | sTNFR2 | 0.487 (<0.0001)** | 1 | 0.225 (0.055) | 0.526 (<0.0001)** | 0.452 (<0.0001)** |

sTNFR 2, soluble tumor necrosis factor receptor 2; sTREM2, soluble triggering receptor expressed on myeloid cells 2. **P* ≤ 0.05. ***P* ≤ 0.01 and False Discovery Rate, p=0.05

**Supplementary Table 3.** Key results of the general linear model with CSF Aβ42, t-tau, and p-tau as the dependent variables among cognitively normal A+ T+ participants from the Alzheimer’s Disease Neuroimaging Initiative cohort

| Model 1: Main effects: CSF sTNFR2 *OR* TREM2 | | | | | | | | | | | | | | | | |  |
| --- | --- | --- | --- | --- | --- | --- | --- | --- | --- | --- | --- | --- | --- | --- | --- | --- | --- |
| Main effect | Dependent variable | | Type III sum of squares | | df | | Mean square | | F | | *P* value | | R^2^ | | Partial eta squared | |  |
| sTNFR2 | Aβ42 | | 0.163 | | 1,12 | | 0.163 | | 0.964 | | 0.345 | | 0.074 | | 0.074 | |  |
|  | t-tau | | 0.039 | | 1,12 | | 0.039 | | 0.464 | | 0.509 | | 0.037 | | 0.037 | |  |
|  | p-tau | | 0.033 | | 1,12 | | 0.033 | | 0.274 | | 0.61 | | 0.022 | | 0.022 | |  |
| sTREM2 | Aβ42 | | 0.038 | | 1,12 | | 0.038 | | 0.214 | | 0.652 | | 0.017 | | 0.017 | |  |
|  | t-tau | | 0.399 | | 1,12 | | 0.399 | | 7.34 | | 0.019* | | 0.38 | | 0.38 | |  |
|  | p-tau | | 0.623 | | 1,12 | | 0.623 | | 8.792 | | 0.012* | | 0.423 | | 0.423 | |  |
| Model 2: Main effects: CSF sTNFR2 *AND* sTREM2 | | | | | | | | | | | | | | | | | |
| Main effect | | Dependent variable | | Type III sum of squares | | df | | Mean square | | F | | *P* value | | R^2^ | | Partial eta squared | |
| sTNFR2 | | Aβ42 | | 0.227 | | 1,11 | | 0.227 | | 1.295 | | 0.279 | | 0.121 | | 0.105 | |
|  |  | t-tau | | 0.001 | | 1,11 | | 0.001 | | 0.01 | | 0.922 | | 0.38 | | 0.001 | |
|  |  | p-tau | | 0.001 | | 1,11 | | 0.001 | | 0.019 | | 0.894 | | 0.424 | | 0.002 | |
| sTREM2 | | Aβ42 | | 0.102 | | 1,11 | | 0.102 | | 0.583 | | 0.461 | | 0.121 | | 0.05 | |
|  |  | t-tau | | 0.36 | | 1,11 | | 0.36 | | 6.084 | | 0.031* | | 0.38 | | 0.356 | |
|  |  | p-tau | | 0.592 | | 1,11 | | 0.592 | | 7.665 | | 0.018* | | 0.424 | | 0.411 | |

CSF, cerebrospinal fluid; df, degrees of freedom; sTNFR 2, soluble tumor necrosis factor receptor 2; sTREM2, soluble triggering receptor expressed on myeloid cells 2. **P* ≤ 0.05. ** *P* ≤ 0.01 and False Discovery Rate, p=0.05

**Supplementary Table 4.** Key results of the general linear model with CSF Aβ42, t-tau, and p-tau as the dependent variables among cognitively normal A+ T– participants from the Alzheimer’s Disease Neuroimaging Initiative cohort

| Model 1: Main effects: CSF sTNFR2 *OR* sTREM2 | | | | | | | | |
| --- | --- | --- | --- | --- | --- | --- | --- | --- |
| Main effect | Dependent variable | Type III sum of squares | df | Mean square | F | *P* value | R^2^ | Partial eta squared |
| sTNFR2 | Aβ42 | 0.027 | 1,9 | 0.027 | 0.17 | 0.69 | 0.018 | 0.018 |
|  | t-tau | 0.28 | 1,9 | 0.28 | 3.031 | 0.11 | 0.25 | 0.25 |
|  | p-tau | 0.27 | 1,9 | 0.26 | 2.47 | 0.15 | 0.21 | 0.21 |
| sTREM2 | Aβ42 | 0.001 | 1,9 | 0.001 | 0.003 | 0.96 | 0 | 0 |
|  | t-tau | 0.15 | 1,9 | 0.15 | 1.42 | 0.26 | 0.14 | 0.17 |
|  | p-tau | 0.201 | 1,9 | 0.2 | 1.77 | 0.22 | 0.16 | 0.16 |
| Model 2: Main effects: CSF sTNFR2 *AND* sTREM2 | | | | | | | | |
| Main effect | Dependent variable | Type III sum of squares | df | Mean square | F | *P* value | R^2^ | Partial eta squared |
| sTNFR2 | Aβ42 | 0.029 | 1,8 | 0.029 | 0.16 | 0.704 | 0.019 | 0.019 |
|  | t-tau | 0.17 | 1,8 | 0.17 | 1.69 | 0.23 | 0.287 | 0.174 |
|  | p-tau | 0.14 | 1,8 | 0.14 | 1.19 | 0.307 | 0.27 | 0.129 |
| sTREM2 | Aβ42 | 0.002 | 1,8 | 0.002 | 0.011 | 0.92 | 0.019 | 0.001 |
|  | t-tau | 0.039 | 1,8 | 0.039 | 0.39 | 0.548 | 0.287 | 0.047 |
|  | p-tau | 0.072 | 1,8 | 0.072 | 0.66 | 0.449 | 0.27 | 0.074 |

CSF, cerebrospinal fluid; df, degrees of freedom; sTNFR 2, soluble tumor necrosis factor receptor 2; sTREM2, soluble triggering receptor expressed on myeloid cells 2.

**Supplementary Table 5.** Key results of the general linear model results with CSF Aβ42, t-tau, and p-tau as the dependent variables among cognitively normal A– T+ participants from the Alzheimer’s Disease Neuroimaging Initiative cohort

| Model 1: Main effects: CSF sTNFR2 *OR* sTREM2 | | | | | | | | | |
| --- | --- | --- | --- | --- | --- | --- | --- | --- | --- |
| Main effect | Dependent variable | | Type III sum of squares | df | Mean square | F | *P* value | R^2^ | Partial eta squared |
| sTNFR2 | Aβ42 | | 0.317 | 1,10 | 0.317 | 4.17 | 0.068 | 0.294 | 0.294 |
|  | t-tau | | 0.353 | 1,10 | 0.353 | 12.997 | 0.005** | 0.565 | 0.565 |
|  | p-tau | | 0.248 | 1,10 | 0.248 | 6.058 | 0.034* | 0.377 | 0.377 |
| sTREM2 | Aβ42 | | 0.002 | 1,10 | 0.002 | 0.017 | 0.898 | 0.002 | 0.002 |
|  | t-tau | | 0.073 | 1,10 | 0.073 | 1.334 | 0.275 | 0.118 | 0.118 |
|  | p-tau | | 0.054 | 1,10 | 0.054 | 0.905 | 0.364 | 0.083 | 0.083 |
| Model 2: Main effects: CSF sTNFR2 *AND* sTREM2 | | | | | | | | | |
| Main effect | Dependent variable | Type III sum of squares | | df | Mean square | F | *P* value | R^2^ | Partial eta squared |
| sTNFR2 | Aβ42 | 0.407 | | 1,9 | 0.407 | 5.496 | 0.044* | 0.38 | 0.379 |
|  | t-tau | 0.282 | | 1,9 | 0.282 | 9.463 | 0.013* | 0.57 | 0.513 |
|  | p-tau | 0.195 | | 1,9 | 0.195 | 4.3 | 0.068 | 0.379 | 0.323 |
| sTREM2 | Aβ42 | 0.092 | | 1,9 | 0.092 | 1.248 | 0.293 | 0.38 | 0.122 |
|  | t-tau | 0.003 | | 1,9 | 0.003 | 0.099 | 0.76 | 0.57 | 0.011 |
|  | p-tau | 0.001 | | 1,9 | 0.001 | 0.032 | 0.862 | 0.379 | 0.004 |

CSF, cerebrospinal fluid; df, degrees of freedom; sTNFR 2, soluble tumor necrosis factor receptor 2; sTREM2, soluble triggering receptor expressed on myeloid cells 2. **P* ≤ 0.05. ** *P* ≤ 0.01 and False Discovery Rate, p=0.05

**Supplementary Table 6.** Key results of the general linear model results with CSF Aβ42, t-tau, and p-tau as the dependent variables among cognitively normal A– T– participants from the Alzheimer’s Disease Neuroimaging Initiative cohort

| Model 1: Main effects: CSF sTNFR2 *OR* sTREM2 | | | | | | | | | |
| --- | --- | --- | --- | --- | --- | --- | --- | --- | --- |
| Main effect | Dependent variable | Type III sum of squares | df | Mean square | F | *P* value | R^2^ | | Partial eta squared |
| sTNFR2 | Aβ42 | 0.058 | 1,33 | 0.058 | 1.24 | 0.27 | 0.036 | | 0.036 |
|  | t-tau | 0.36 | 1,33 | 0.36 | 5.66 | 0.023* | 0.15 | | 0.146 |
|  | p-tau | 0.203 | 1,33 | 0.203 | 3.36 | 0.076 | 0.092 | | 0.092 |
| sTREM2 | Aβ42 | 0.077 | 1,33 | 0.077 | 1.68 | 0.2 | 0.05 | | 0.049 |
|  | t-tau | 0.31 | 1,33 | 0.31 | 4.89 | 0.034* | 0.13 | | 0.129 |
|  | p-tau | 0.16 | 1,33 | 0.16 | 2.54 | 0.12 | 0.071 | | 0.071 |
| Model 2: Main effects: CSF sTNFR2 *AND* sTREM2 | | | | | | | | | |
| Main effect | Dependent variable | Type III sum of squares | df | Mean square | F | *P* value | R^2^ | | Partial eta squared |
| sTNFR2 | Aβ42 | 0.01 | 1 | 0.01 | 0.212 | 0.649 | 0.68 | | 0.007 |
|  | t-tau | 0.411 | 1 | 0.411 | 8.257 | 0.007* | | 0.36 | 0.21 |
|  | p-tau | 0.248 | 1 | 0.248 | 4.514 | 0.042* | 0.23 | | 0.127 |
| sTREM2 | Aβ42 | 0.00006 | 1 | 0.00006 | 0.001 | 0.972 | 0.68 | | 0.00001 |
|  | t-tau | 0.154 | 1 | 0.154 | 3.085 | 0.089 | 0.36 | | 0.091 |
|  | p-tau | 0.103 | 1 | 0.103 | 1.87 | 0.181 | 0.23 | | 0.057 |
| sTNFR2 * sTREM2 | Aβ42 | 0.009 | 1 | 0.009 | 0.185 | 0.67 | 0.68 | | 0.006 |
|  | t-tau | 0.388 | 1 | 0.388 | 7.794 | 0.009** | 0.36 | | 0.201 |
|  | p-tau | 0.234 | 1 | 0.234 | 4.26 | 0.047* | 0.23 | | 0.121 |

CSF, cerebrospinal fluid; df, degrees of freedom; sTNFR 2, soluble tumor necrosis factor receptor 2; sTREM2, soluble triggering receptor expressed on myeloid cells 2. **P* ≤ 0.05. ** *P* ≤ 0.01 and False Discovery Rate, p=0.05

**Supplementary Table 7.** Key results of the linear mixed-effects regression model with CDR-SB as the dependent variable among A+ T+ cognitively normal participants from the Alzheimer’s Disease Neuroimaging Initiative cohort (Model 3)

| Parameter | Estimate | Standard error | df | t | *P* value | 95% confidence interval | | FDR |
| --- | --- | --- | --- | --- | --- | --- | --- | --- |
|  |  |  |  |  |  | Lower bound | Upper bound |  |
| Intercept | ‒9.41246 | 15.49706 | 13.616 | ‒0.607 | 0.554 | ‒42.7385 | 23.91355 | 0.89 |
| sTREM2 | ‒0.5698 | 1.318155 | 15.784 | ‒0.432 | 0.671 | ‒3.36728 | 2.227671 | 0.89 |
| sTNFR2 | ‒1.09284 | 5.436051 | 13.3 | ‒0.201 | 0.844 | ‒12.8098 | 10.62414 | 0.84 |
| t-tau | 1.888528 | 2.171067 | 12.782 | 0.87 | 0.4 | ‒2.80991 | 6.586963 | 1 |
| visit number in years | 2.162563 | 3.412962 | 18.679 | 0.634 | 0.534 | ‒4.98917 | 9.314298 | 1 |
| visit number in years × sTREM2 | 0.948695 | 0.389292 | 21.935 | 2.437 | 0.023* | 0.141213 | 1.756177 | 0.092 |
| visit number in years × sTNFR2 | ‒0.45023 | 1.361512 | 17.895 | ‒0.331 | 0.745 | ‒3.31186 | 2.411404 | 0.85 |
| visit number in years × t-tau | ‒1.61154 | 0.482982 | 18.241 | ‒3.337 | 0.004* | ‒2.62529 | ‒0.5978 | 0.032* |

CSF sTREM2 × visit number + CSF sTNFR2 × visit number, CSF t-tau × visit number + CSF sTREM2 + CSF sTNFR2 + CSF t-tau + visit number (fixed effect). CDR-SB, Clinical Dementia Rating–Sum of Boxes; CSF, cerebrospinal fluid; df, degrees of freedom; FDR, false discovery rate; sTNFR 2, soluble tumor necrosis factor receptor 2; sTREM2, soluble triggering receptor expressed on myeloid cells 2. **P* ≤ 0.05.

**Supplementary Table 8.** Key results of the linear mixed-effects regression model with CDR-SB as the dependent variable among CN A– T+ cognitively normal participants from the Alzheimer’s Disease Neuroimaging Initiative cohort (Model 3)

| Parameter | Estimate | Stanrdard error | df | t | *P* value | 95% confidence interval | | FDR |
| --- | --- | --- | --- | --- | --- | --- | --- | --- |
|  |  |  |  |  |  | Lower bound | Upper bound |  |
| Intercept | ‒11.5566 | 14.10101 | 20.912 | ‒0.82 | 0.422 | ‒40.8888 | 17.77554 | 0.68 |
| sTREM2 | 0.104425 | 0.303009 | 20.623 | 0.345 | 0.734 | ‒0.52642 | 0.735269 | 0.73 |
| sTNFR2 | ‒2.07991 | 3.013446 | 20.894 | ‒0.69 | 0.498 | ‒8.34866 | 4.188838 | 0.57 |
| t-tau | 1.217393 | 1.587566 | 21.038 | 0.767 | 0.452 | ‒2.08377 | 4.518553 | 0.6 |
| visit number in years | 6.367836 | 2.248418 | 32.502 | 2.832 | 0.008* | 1.790729 | 10.94494 | 0.064 |
| visit number in years × TREM2 | ‒0.09227 | 0.047067 | 31.468 | ‒1.96 | 0.059 | ‒0.1882 | 0.003667 | 0.12 |
| visit number in years × TNFR2 | 1.238786 | 0.479374 | 32.571 | 2.584 | 0.014* | 0.263004 | 2.214568 | 0.056 |
| visit number in years × t-tau | ‒0.61087 | 0.25609 | 32.983 | ‒2.385 | 0.023* | ‒1.1319 | ‒0.08985 | 0.061 |

CSF sTREM2 × visit number + CSF sTNFR2 × visit number, CSF t-tau × visit number + CSF sTREM2 + CSF sTNFR2 + CSF t-tau + visit number (fixed effect). CDR-SB, Clinical Dementia Rating–Sum of Boxes; CSF, cerebrospinal fluid; df, degrees of freedom; FDR, false discovery rate; sTNFR 2, soluble tumor necrosis factor receptor 2; sTREM2, soluble triggering receptor expressed on myeloid cells 2. **P* ≤ 0.05.

**Supplementary Table 9.** Key results of the linear mixed-effects regression model with CDR-SB as the dependent variable among A+ T– cognitively normal participants from the Alzheimer’s Disease Neuroimaging Initiative cohort (Model 3)

| Parameter | Estimate | Standard error | df | t | *P* value | 95% confidence interval | | FDR |
| --- | --- | --- | --- | --- | --- | --- | --- | --- |
|  |  |  |  |  |  | Lower bound | Upper bound |  |
| Intercept | ‒1.3865 | 26.11812 | 7.598 | ‒0.053 | 0.959 | ‒62.174 | 59.401 | 1 |
| sTREM2 | 0.148079 | 0.851917 | 7.653 | 0.174 | 0.867 | ‒1.83204 | 2.1282 | 1 |
| sTNFR2 | ‒0.05622 | 4.984667 | 7.623 | ‒0.011 | 0.991 | ‒11.6506 | 11.53818 | 0.98 |
| t-tau | 0.040514 | 5.698849 | 7.607 | 0.007 | 0.995 | ‒13.2202 | 13.30128 | 1 |
| visit number in years | ‒0.53 | 2.781414 | 64.132 | ‒0.191 | 0.849 | ‒6.08629 | 5.026297 | 0.12 |
| visit number in years × TREM2 | 0.018807 | 0.116185 | 69.169 | 0.162 | 0.872 | ‒0.21297 | 0.250579 | 1 |
| visit number in years × TNFR2 | ‒0.64911 | 0.607689 | 67.164 | ‒1.068 | 0.289 | ‒1.86201 | 0.563791 | 1 |
| visit number in years × t-tau | ‒0.39686 | 0.636389 | 65.071 | ‒0.624 | 0.535 | ‒1.66779 | 0.874072 | 0.12 |

CSF sTREM2 × visit number + CSF sTNFR2 × visit number, CSF t-tau × visit number + CSF sTREM2 + CSF sTNFR2 + CSF t-tau + visit number (fixed effect). CDR-SB, Clinical Dementia Rating–Sum of Boxes; CSF, cerebrospinal fluid; df, degrees of freedom; FDR, false discovery rate; sTNFR 2, soluble tumor necrosis factor receptor 2; sTREM2, soluble triggering receptor expressed on myeloid cells 2.

**Supplementary Table 10.** Key results of the linear mixed-effects regression model with CDR-SB as the dependent variable among A– T– cognitively normal participants from the Alzheimer’s Disease Neuroimaging Initiative cohort (Model 3)

| Parameter | Estimate | Standard error | df | t | *P* value | 95% confidence interval | | FDR |
| --- | --- | --- | --- | --- | --- | --- | --- | --- |
|  |  |  |  |  |  | Lower bound | Upper bound |  |
| Intercept | 0.557958 | 3.288641 | 58.561 | 0.17 | 0.866 | ‒6.02363 | 7.139545 | 1 |
| sTREM2 | ‒0.0432 | 0.144865 | 59.059 | ‒0.298 | 0.767 | ‒0.33307 | 0.246665 | 1 |
| sTNFR2 | ‒0.11261 | 0.744092 | 63.811 | ‒0.151 | 0.88 | ‒1.59919 | 1.373966 | 1 |
| t-tau | ‒0.00878 | 0.424734 | 59.353 | ‒0.021 | 0.984 | ‒0.85857 | 0.841002 | 0.98 |
| visit number in years | 0.35342 | 0.552736 | 121.189 | 0.639 | 0.524 | ‒0.74085 | 1.447689 | 1 |
| visit number in years × TREM2 | 0.051434 | 0.02551 | 113.111 | 2.016 | 0.046 | 0.000894 | 0.101974 | 0.37 |
| visit number in years × TNFR2 | 0.250357 | 0.153406 | 156.433 | 1.632 | 0.105 | ‒0.05266 | 0.553371 | 0.42 |
| visit number in years × t-tau | ‒0.11179 | 0.074633 | 121.036 | ‒1.498 | 0.137 | ‒0.25954 | 0.035968 | 0.37 |

CSF sTREM2 × visit number + CSF sTNFR2 × visit number, CSF t-tau × visit number + CSF sTREM2 + CSF sTNFR2 + CSF t-tau + visit number (fixed effect). CDR-SB, Clinical Dementia Rating–Sum of Boxes; CSF, cerebrospinal fluid; df, degrees of freedom; FDR, false discovery rate; sTNFR 2, soluble tumor necrosis factor receptor 2; sTREM2, soluble triggering receptor expressed on myeloid cells 2.

**Supplementary Table 11.** Demographics of A+T+ participants with CSF sTREM2 values (includes those without concomitant sTNFR2 data) along with CSF AD biomarkers in the Alzheimer’s Disease Neuroimaging Initiative cohort for sensitivity analysis

| Demographic variable | CN A+T+  (*n* = 16) | MCI A+T+  (*n* = 111) | Dementia A+T+  (*n* = 57) |
| --- | --- | --- | --- |
|  | Mean (SD) | Mean (SD) | Mean (SD) |
| Age, y | 78.41 (5.14) | 73.69(7.2) | 73.541 (8.0) |
| Sex (% female) | 43.8% | 42.3% | 49.1% |
| *APOEε4* (%) | 62.5% | 70.3% | 80.7% |
| Patient education, y | 16.88 (2.68) | 15.44(3.0) | 15.07 (3.18) |
| Baseline CDR-SB score | 0.00(0.00) | 1.65 (0.91) | 4.26 (1.43) |
| Log_2_ Aβ42 | 9.41 (0.39) | 9.25 (0.38) | 9.07 (0.44) |
| Log_2_ t-tau | 8.36 (0.35) | 8.46 (0.41) | 8.51 (0.41) |
| Log_2_ p-tau | 5.05 (0.40) | 5.16 (0.46) | 5.21 (0.46) |
| Log_2_ sTREM2 | 12.41 (0.57) | 11.98(0.71) | 11.98 (0.61) |

CDR-SB, Clinical Dementia Rating–Sum of Boxes; SD, standard deviation; sTREM2, soluble triggering receptor expressed on myeloid cells 2

**Supplementary Table 12.** Demographics of cognitively normal participants with CSF sTREM2 values (includes those without concomitant sTNFR2 data) along with CSF AD biomarkers in the Alzheimer’s Disease Neuroimaging Initiative cohort for sensitivity analysis

| Demographic variable | A– T– (*n* = 40) | A+ T+ (*n* = 16) | A+ T– (*n* = 14) | A– T+ (*n* = 16) |
| --- | --- | --- | --- | --- |
|  | Mean (SD) | Mean (SD) | Mean (SD) | Mean (SD) |
| Age, y | 74.68(4.55) | 78.41 (5.14) | 73.76 (5.43) | 76.74 (5.63) |
| Sex (% female) | 40% | 43.8% | 42.9% | 56.3% |
| *APOEε4* (%) | 11.4% | 62.5% | 35.7% | 6.3% |
| Patient education, y | 15.25(2.92) | 16.88 (2.68) | 15.57 (4.1) | 15.88 (2.5) |
| Baseline CDR-SB score | 0.01 (0.79) | 0.00(0.00) | 0.07 (0.18) | 0.03(0.13) |
| Log_2_ Aβ42 | 10.47 (0.21) | 9.41 (0.39) | 9.43 (0.41) | 10.56 (0.28) |
| Log_2_ t-tau | 7.56 (0.26) | 8.36 (0.35) | 7.26 (0.49) | 8.28 (0.22 |
| Log_2_ p-tau | 4.06 (0.25) | 5.05 (0.40) | 3.82 (0.47) | 4.80 (0.22) |
| Log_2_ sTREM2 | 11.93 (0.74) | 12.41 (0.57) | 11.2 (0.80) | 12.39 (0.87) |

CDR-SB, Clinical Dementia Rating–Sum of Boxes; SD, standard deviation; sTREM2, soluble triggering receptor expressed on myeloid cells 2.

**Supplementary Table 13.** Sensitivity analysis of linear mixed-effects regression model with CDR-SB as the dependent variable among participants with A+ T+ mild cognitive impairment from the Alzheimer’s Disease Neuroimaging Initiative cohort with n= 111 with sTREM data alone

| Parameter | Estimate | Standard error | df | t | *P* value | 95% confidence interval | |
| --- | --- | --- | --- | --- | --- | --- | --- |
|  |  |  |  |  |  | Lower bound | Upper bound |
| Intercept | 7.06 | 4.33 | 588 | 1.63 | 0.10 | -1.46 | 15.57 |
| sTREM2 | -0.28 | 0.29 | 588 | -0.95 | 0.34 | -0.85 | 0.30 |
| t-tau | 0.26 | 0.50 | 588 | -0.51 | 0.61 | -1.24 | 0.73 |
| visit number in years | -0.17 | 0.99 | 588 | -0.17 | 0.86 | -2.10 | 1.77 |
| visit number in years × sTREM2 | -0.13 | 0.08 | 588 | -1.61 | 0.11 | -0.29 | 0.029 |
| visit number in years × t-tau | 0.29 | 0.13 | 588 | 2.22 | 0.026 | 0.03 | 0.55 |

CSF sTREM2 × visit number + CSF sTNFR2 × visit number, CSF t-tau × visit number + CSF sTREM2 + CSF sTNFR2 + CSF t-tau + visit number (fixed effect). CDR-SB, Clinical Dementia Rating–Sum of Boxes; CSF, cerebrospinal fluid; df, degrees of freedom; sTNFR 2, soluble tumor necrosis factor receptor 2; sTREM2, soluble triggering receptor expressed on myeloid cells 2.

**Supplementary Table 14.** Sensitivity analysis of linear mixed-effects regression model with CDR-SB as the dependent variable among participants with A+ T+ dementia from the Alzheimer’s Disease Neuroimaging Initiative cohort with n= 57 with sTREM data alone

| Parameter | Estimate | Standard error | df | t | *P* value | 95% confidence interval | |
| --- | --- | --- | --- | --- | --- | --- | --- |
|  |  |  |  |  |  | Lower bound | Upper bound |
| Intercept | 10.71 | 7.41 | 159 | 1.44 | 0.15 | -3.92 | 25.36 |
| sTREM2 | 0.10 | 0.52 | 159 | 0.19 | 0.85 | -0.93 | 1.13 |
| t-tau | -0.93 | 0.78 | 159 | -1.19 | 0.24 | -2.48 | 0.62 |
| visit number in years | -0.96 | 5.38 | 159 | -0.18 | 0.86 | -11.58 | 9.65 |
| visit number in years × sTREM2 | -0.76 | 0.38 | 159 | -2.00 | 0.04* | -1.50 | -0.01 |
| visit number in years × t-tau | 1.40 | 0.59 | 159 | 2.36 | 0.02* | 0.23 | 2.57 |

CSF sTREM2 × visit number + CSF sTNFR2 × visit number, CSF t-tau × visit number + CSF sTREM2 + CSF sTNFR2 + CSF t-tau + visit number (fixed effect). CDR-SB, Clinical Dementia Rating–Sum of Boxes; CSF, cerebrospinal fluid; df, degrees of freedom; sTNFR 2, soluble tumor necrosis factor receptor 2; sTREM2, soluble triggering receptor expressed on myeloid cells 2.

**Supplementary Table 15.** Sensitivity analysis of linear mixed-effects regression model with CDR-SB as the dependent variable among participants with A+ T+ CN from the Alzheimer’s Disease Neuroimaging Initiative cohort with n= 16 with sTREM data alone

| Parameter | Estimate | Standard error | df | t | *P* value | 95% confidence interval | | FDR |
| --- | --- | --- | --- | --- | --- | --- | --- | --- |
|  |  |  |  |  |  | Lower bound | Upper bound |  |
| Intercept | -4.19 | 8.92 | 93 | -0.47 | 0.64 | -21.91 | 13.52 | 0.64 |
| sTREM2 | -0.63 | 0.76 | 93 | -0.83 | 0.41 | -2.13 | 0.88 | 0.49 |
| t-tau | 1.32 | 1.21 | 93 | 1.09 | 0.28 | -1.09 | 3.72 | 0.56 |
| visit number in years | 1.44 | 1.62 | 93 | 0.88 | 0.37 | -1.79 | 4.67 | 0.55 |
| visit number in years × sTREM2 | 0.83 | 0.19 | 93 | 4.35 | <0.0001* | 0.45 | 1.22 | 0.0006* |
| visit number in years × t-tau | -1.34 | 0.26 | 93 | -5.10 | <0.0001* | -1.86 | -0.81 | 0.0003* |

CSF sTREM2 × visit number + CSF sTNFR2 × visit number, CSF t-tau × visit number + CSF sTREM2 + CSF sTNFR2 + CSF t-tau + visit number (fixed effect). CDR-SB, Clinical Dementia Rating–Sum of Boxes; CSF, cerebrospinal fluid; df, degrees of freedom; sTNFR 2, soluble tumor necrosis factor receptor 2; sTREM2, soluble triggering receptor expressed on myeloid cells 2.
